# The sociobiome–oral microbiome mediates dental caries among Indigenous Australians

**DOI:** 10.3389/fcimb.2025.1721183

**Published:** 2025-12-17

**Authors:** Sonia Nath, Laura S. Weyrich, Gina Guzzo, Joanne Hedges, Manisha Tamrakar, Kostas Kapellas, Lisa Jamieson

**Affiliations:** 1 Adelaide Dental School, The University of Adelaide, Adelaide, SA, Australia; 2 School of Biological Sciences, University of Adelaide, Adelaide, SA, Australia

**Keywords:** aboriginal health, dental caries, health inequities, oral microbiome, socioeconomic status

## Abstract

**Background:**

The sociobiome refers to the social and socioeconomic conditions that shape human microbial communities, linking structural inequities to biological changes in the microbiome. The aim of this study was to examine how individual and neighbourhood socioeconomic status (SES) are associated with the oral microbiota and dental caries in Indigenous Australian adults.

**Methods:**

This cross-sectional study involved 100 Indigenous Australian participants aged ≥ 18 years and was embedded within a decolonising, community−based participatory research framework. Demographic, socioeconomic, and oral health behaviour data were collected, followed by a dental examination and collection of saliva and plaque samples. The samples were analysed using 16S rRNA amplicon sequencing, and alpha and beta diversity, redundancy analysis, and differential abundance analysis were conducted. Mediation models were used to examine associations between income (Healthcare card ownership), education (≤ secondary), the oral microbiome, and dental caries.

**Results:**

The microbiome analyses showed saliva had higher alpha diversity (p < 0.01), and beta diversity was significantly different between saliva and plaque (adonis p < 0.001). In saliva, alpha diversity was lower with advancing age, secondary education, income, Healthcare card ownership, and dental caries presence. Beta diversity in saliva microbiome composition showed a stronger association with SES than plaque, with income source (R²=3.8%, *p* < 0.01), education (R²=2.0%, *p* < 0.01), and dental caries (R²=2.2%, *p* < 0.01). Differential abundance analysis showed that the *Rikenellaceae RC9 gut group, F0058*, *Fillifactor*, and *Treponema* were elevated in the low-SES and caries groups. Mediation analysis showed that 75% of the impact of low income on caries was mediated via microbiome shifts, compared with 21% for education, highlighting the strong role of oral microbiome alterations in SES-driven caries risk.

**Conclusion:**

Socioeconomic disadvantage is associated with variations in the oral microbiome, and these microbial patterns may explain the link between lower income and dental health caries. Saliva may serve as a sensitive biomarker of socioeconomic gradients in oral health. These findings support integrated approaches that address structural determinants of disadvantage alongside microbiome-informed preventive strategies when tackling oral health inequities in Indigenous populations.

## 1 Introduction

Oral health disparities among Aboriginal and/or Torres Strait Islander Peoples (hereafter respectfully referred to as Indigenous people) represent a profound intersection of historical injustice, structural inequity, and biological vulnerability (Jamieson et al., 2021b). Indigenous Australians experience disproportionately high rates of dental caries, periodontal disease, and tooth loss compared to non-Indigenous Australian populations (Nath et al., 2021b, 2022). These inequities are not a reflection of individual behaviour but are deeply rooted in the enduring legacies of colonisation and systemic racism that perpetuate socioeconomic disadvantage and restrict access to culturally safe oral healthcare (Jamieson et al., 2021b). Broader policy frameworks, including neoliberal approaches that emphasise privatised dental care and reduced public health funding, continue to influence access to culturally safe, preventive oral healthcare for Indigenous populations (Jamieson et al., 2021a).

The “sociobiome” concept describes the way social and socioeconomic factors, at both individual and community levels, shape the composition, diversity, and function of the human microbiome, leading to shared microbial characteristics among people with similar exposures or circumstances (Nobre and Alpuim Costa, 2022; Kwak et al., 2024). Unlike traditional approaches that focus solely on genetics or individual behaviour, the sociobiome framework examines how upstream social determinants such as income, education, neighbourhood environment, and public policy shape shared lived experiences that directly and indirectly impact microbial communities in the body, including oral and gut microbiota (Nobre and Alpuim Costa, 2022; Zuniga-Chaves et al., 2023). This concept recognises that marginalised populations not only experience increased risk due to their environments but also display distinct microbial signatures that contribute to disease development.

Within the World Health Organization framework, five key social determinants of health, income security and social protection, education, employment and working conditions, social inclusion, and access to health services are central pathways through which political and economic systems shape health (World Health Organization, 2024). Lower educational attainment and income are linked to elevated abundances of caries-associated taxa, such as *Lactobacillus* and *Prevotella* (Miller et al., 2016; Kwak et al., 2024). Neighbourhood-level factors, such as living in a remote location or a lower socioeconomic suburb, often exhibit reduced microbial diversity, increased abundance of cariogenic taxa, and altered metabolic function, which heighten caries risk (Handsley-Davis et al., 2021, 2022).

Researchers found that Indigenous Australians had significantly higher oral microbial diversity and distinct composition than non-Indigenous individuals, including unique taxa not previously observed in the human oral microbiota (Handsley-Davis et al., 2021, 2022). Similarly, work in Venezuela, the Philippines, and Uganda has reported systematic differences between the oral microbiota of Indigenous groups with traditional lifestyles and counterparts living agricultural or industrialised lifestyles (Handsley-Davis et al., 2020). Despite growing recognition, the extent to which SES influences these microbial shifts remains poorly understood, and limited research has compared how saliva and plaque samples capture these effects differently (Kageyama et al., 2017). Therefore, the aim of this study is to investigate how individual (e.g., education level, income source, ownership of healthcare card, dental affordability) and neighbourhood-level socioeconomic factors shape the composition and diversity of the oral microbiome in Indigenous Australian populations. Understanding these relationships is essential for addressing both the biological mechanisms of disease and the upstream structural inequities that perpetuate oral health disparities in Indigenous Australians.

## 2 Methods

This finding of this study is part of a larger mixed-method study with a 12-month follow-up. A detailed study protocol has been published (Jamieson et al., 2023). Ethical approval was obtained by the Indigenous Health Council of South Australia’s Human Research Ethics Committee and the University of Adelaide Human Research Ethics Committee (Combined number #04-22-990). The research was conducted in accordance with the World Medical Association Declaration of Helsinki. All participants received written and verbal information about the study, and written informed consent was obtained.

### 2.1 Positionality statement

We acknowledge the importance of positionality, and this study was conducted in strict accordance with the CONSIDER statement and Aboriginal and Torres Strait Islander research ethics guidelines, embedding Indigenous governance and accountability throughout (Huria et al., 2019). This project adopted a decolonising methodology and a collaborative, community-based participatory action research approach, integrating Indigenous and Western research methods into a co-designed framework (Pham et al., 2025). This study was overseen by the Indigenous Oral Health Unit Reference Group (IOHURG), which spanned all stages, including study design, data collection, analysis, interpretation, employment of project staff, and the cultural appropriateness of language. Our team consisted of Indigenous and non-Indigenous researchers (including the first and last authors) with extensive experience, bringing diverse backgrounds. Each member critically evaluated how their own backgrounds influenced their understanding of healthcare access and privilege within the Australian health system. Key to this process was the mentorship by a senior Indigenous researcher (JH) and consistent dialogue with the IOHURG and community leaders. The findings were shared with participating communities through co-created, culturally appropriate formats, including community presentations, plain-language summaries, and visual infographics, with opportunities for dialogue and further input before wider dissemination.

### 2.2 Study design and data collection

The data collection took place from July 2022 to December 2023, followed by a 12-month follow-up phase from March 2024 to March 2025. This research was conducted in South Australia, on the traditional lands of the Kaurna (Greater Adelaide), Adnyamathanha (Hawker), Nukunu (Port Augusta), Barngarla (Port Augusta), and Ngarrindjeri (Murray Bridge) Peoples. This study analysed baseline data from 100 adults (with microbial samples collected) from a convenience sample of 280 adult participants. Based on previous research (Jamieson et al., 2019; Hedges et al., 2023), recruitment methods included self-nomination, word of mouth, flyers and posters, and personal visits to local community centres and health clinics. Eligibility criteria included participants being Indigenous or Torres Strait Islander, or both, over 18 years of age, and living in South Australia.

Data collection and dental examinations were conducted in participants’ homes, workplaces, community centres, or dental clinics, with each participant’s most convenient and comfortable setting used. Data were collected by experienced Indigenous and non-Indigenous research officers, all of whom were trained and calibrated (Jamieson et al., 2023). Indigenous research officers administered self-reported questionnaires, which were completed as interviews, on paper, or online via REDCap (Research Electronic Data Capture) software (Harris et al., 2009). The structured questionnaire was adapted from instruments previously used in Indigenous Australian oral health surveys, which have demonstrated acceptable reliability and construct validity for sociodemographic, service-use and self−reported oral health measures (Jamieson et al., 2020, 2023). Formal psychometric testing was not repeated for this study.

### 2.3 Dental examination

The dental practitioners (SN & KK) performed comprehensive oral assessments using head-LED lights, portable dental equipment, and single-use dental instruments. The examination included a five-surface evaluation of each tooth, employing a coding system to record each tooth as either sound (S) or decayed (D) using International Caries Detection and Assessment System (ICDAS) II scores. Participants with an ICDAS II score of 4 or higher were categorised as having caries present (Iranzo-Cortés et al., 2013). Before data collection, both dental practitioners (SN and KK) underwent standardised calibration exercises involving duplicate dental examinations on 10 participants not included in this study. Inter-examiner reliability was assessed using Cohen’s kappa statistic, with a kappa value of 0.89 (95% CI: 0.82–0.95) for ICDAS II caries detection, indicating excellent agreement.

### 2.4 Variables

The categorisation of each variable is described in detail in **Supplementary Text S1** and consists of five domains. The sociodemographic domain included age, sex, and residential location. The SES domain included both individual-level and neighbourhood-level indicators. Four measures were considered for individual-level SES: education level (secondary or tertiary), income source (job or Centrelink), ownership of a Healthcare card (HCC) and difficulty paying $100 for dental treatment (hard or not hard). Centrelink income refers to money received from Australian Government social security payments and benefits (such as pensions, allowances, or welfare support). HCC ownership provides eligibility for cheaper prescription medicines and various health and cost-of-living concessions based on low income or qualifying benefits. Neighbourhood-level SES was assessed using the Socio-Economic Indexes for Areas (SEIFA) and remoteness score (Australian Bureau of Statistics (Jul2021-Jun2026); Australian Bureau of Statistics, 2021). The knowledge, attitude and beliefs domain included time since the last dental visit (<1 year or >1 year), the reason for visiting the dentist (check-up or problem-based), and cost-related avoidance of dental visits (yes or no). The health behaviour factors included self-reported diabetes, smoking habits (current, past, or non-smoker) and alcohol consumption and frequency (never, monthly, or weekly). Oral health outcomes factors included self-rated oral health (good or poor) and dental caries status (present or absent).

### 2.5 Sample collection

Saliva and plaque samples were collected by trained and calibrated dental practitioners (SN & KK). Unstimulated 2 ml saliva samples were collected using a saliva collection tube (Zymo DNA/RNA Shield Saliva Collection Kit, Zymo Research, California, USA). The supragingival plaque was collected from six pooled sites using a sterile single-use universal Gracey curette following a standardised protocol (Nath et al., 2021c) and carefully transferred into a 2 mL tube containing a DNA/RNA shield (Zymo Research, California, USA) to stabilise nucleic acids and prevent microbial degradation. These sites were selected to capture both posterior and anterior tooth surfaces across quadrants, providing a composite plaque sample that reflects overall supragingival biofilm.

Additional control samples were collected to monitor environmental and instrument-related contamination. Environmental (ENV) controls consisted of open collection tubes exposed to the sampling environment during each session, and curette wash (CW) controls by rinsing unused sterile curettes in DNA/RNA Shield. These controls were processed and sequenced alongside biological samples to aid in the identification of contaminants. Samples were stored in portable coolers with ice packs during transport and transferred to laboratory freezers within 4–6 hours of collection. Maximum storage time at -20 °C prior to shipment to the Pennsylvania State University ranged from 2 to 18 months. All the samples were then shipped to the MicroARCH Lab at Pennsylvania State University for 16S rRNA amplicon sequencing. This facility is designed to reduce contaminant signatures using specific workflows, UV radiation, and positive pressure. All work was conducted in this laboratory using a still-air hood. The entire protocol for biological and control sample collection is described in **Supplementary Text S2**.

### 2.6 Microbial analysis and data pre-processing:

DNA was extracted from all the biological samples using the MagMax Microbiome Ultra Nucleic Acid Isolation Kit (Applied Biosystems). Extraction blank controls (EBCs) and no template controls (NTCs) were also processed alongside to identify background laboratory contamination. The V4 region of the 16S rRNA gene was amplified, purified, and sequenced on an Illumina MiSeq platform, as previously described (Adler et al., 2013). This method has been previously used to amplify Indigenous Australian oral microbiomes using the 16S rRNA V4 region, with special attention paid to contaminant control, inclusion of contaminant monitoring, and clean-room protocols (Handsley-Davis et al., 2022). **Supplementary Text S3** describes the complete DNA extraction, amplification, and sequencing process, as previously adapted from the Caporaso method (Caporaso et al., 2012). Data pre-processing was performed in QIIME2 software (v.2024.11) (Bolyen et al., 2019), which involved demultiplexing, quality filtering, and denoising reads to generate amplicon sequence variants (ASVs) (Adler et al., 2013). Sequences were aligned with MAFFT to create phylogenetic trees and a rooted phylogenetic tree. The Silva 138 (99% out full-length sequences) database was used for taxonomic assignment to ASVs. The detailed protocol for bioinformatics analysis is outlined in **Supplementary Text S4**.

Contaminant removal was performed in two sequential steps for comprehensive contaminant detection and removal using *decontam* (Davis et al., 2018). First, we compared EBC (n=40) and NTC (n=6) samples with biological samples (**Supplementary Figures S1** and **S2**) and identified 54 taxa that were more abundant in controls and biological samples (**Supplementary Table S1**); all of these taxa were removed from the dataset. Next, we compared ASVs within the ENV (n=20) and CW (n=2) controls (**Supplementary Figure S2**) and detected an additional 39 contaminants (**Supplementary Table S2**); these taxa were also removed from the data set. After both decontamination steps, microbiota counts, metadata, and taxonomy artefacts were imported into RStudio to create a *phyloseq* object for further analysis. Data filtering was performed in multiple stages to refine the microbiome dataset. Initially, ASVs with zero counts across all samples were removed. Subsequently, rare taxa were filtered out based on prevalence (<5% of samples) or total abundance (<10 reads), along with known contaminant sequences identified as chloroplast or mitochondria. Saliva samples had sequencing depths ranging from 17,692 to 95,253 reads, with a median of 56,456 and a mean of 55,005. Plaque samples exhibited sequencing depths ranging from 12,362 to 88,115 reads, with a median of 62,042 and a mean of 60,140. After filtering, saliva and plaque samples contained 678 and 544 ASVs.

### 2.7 Statistical analysis

#### 2.7.1 Comparison of saliva and plaque samples

All the analysis was conducted in R version 4.4.2 (2024-10-31), and the script can be found at https://github.com/sonianath/Indigenous_Australian_Oral_Microbiome. Alpha diversity measures of the microbiome were analysed for two sample types: plaque and saliva, after the data were rarefied to even sampling depth, where the rarefaction curve reached a plateau (**Supplementary Figure S3**). The metrics used were “Observed” (number of observed taxa) and Shannon’s diversity (a measure of microbial diversity that incorporates both richness and evenness). Violin plots were generated to visualise the distribution of alpha diversity for each sample type and compared using the Wilcoxon rank-sum test. Beta diversity was visualised using non-metric multidimensional scaling (NMDS) in the *MicroViz* package (Barnett et al., 2021) based on Aitchison’s distances. A compositional bar plot was generated to visualise the relative abundance of microbial genera in plaque and saliva samples aggregated at the genus level, retaining the top 12 most abundant genera. Saliva and plaque samples were compared for core microbiome genera, defined as those present in at least 80% of samples with a minimum abundance of 5 counts. The core genera unique to saliva and plaque, and shared between the two sample types, were determined, and a Venn diagram was created (Chen and Boutros, 2011). Lastly, differentially abundant bacterial genera were identified in saliva and plaque samples, controlling for age and sex using the negative binomial model for count data and cumulative sum scaling (CSS) normalisation from the *MaAsLin2* package (Mallick et al., 2021).

#### 2.7.2 Analysing the impact of socioeconomic factors on the oral microbiome from the plaque and saliva samples

Redundancy analysis (RDA) was used to assess relationships between microbial community composition and explanatory variables using the *MicroViz* package (Barnett et al., 2021). Explanatory variables included demographic factors (e.g., age categories, female gender), socioeconomic indicators (e.g., low SES, secondary education or less, Centrelink/social welfare as an income source, healthcare card ownership, having difficulty paying $100 for dental treatment), behavioural factors (e.g., smoking, alcohol consumption) and oral health factors (poor self-rated oral health, dental caries. The ordination plot was generated to visualise the clustering of samples and the directional influence of variables on microbial community composition grouped by age categories (18–34 years & 35–54 years), with taxa and explanatory variables represented as vectors, with their length and direction indicating their strength and influence (DeSarbo et al., 2016). Similarly, the alpha diversity (Observed and Shannon’s) and beta diversity (Aitchison’s distance) were compared separately across independent variables for plaque and saliva samples after rarefaction to even depth. For alpha diversity, either the Wilcoxon rank-sum test for two groups or the Kruskal-Wallis test for multiple groups was used, followed by the *post hoc* Dunn test for pairwise comparisons, controlling for false discovery rates using the Benjamini-Hochberg method (p<0.05). For beta diversity, NMDS plots were used for comparison, and pairwise PERMANOVA was used to test differences between groups. A multivariate PERMANOVA included sociodemographic, SES, knowledge, attitude, and health behaviour variables, and these variables were compared to explain the variance of each variable. Only the SES variables that were significant in beta diversity were analysed for differential abundance, along with dental caries (only for saliva samples), controlling for age and sex (Mallick et al., 2021).

### 2.8 Mediation analysis

Mediation analysis was used to examine how income source (Centrelink *vs*. Job) and education level (secondary *vs*. tertiary) affect dental caries, with the oral microbiome as a potential mediator. Covariates included in the multivariate models were selected based on: (1) significant univariate associations with outcome variables; and (2) theoretical relevance within the sociobiome framework. For propensity score matching in mediation analysis, covariates were selected to balance potential confounders between exposure groups, including demographic factors (age, sex), socioeconomic indicators (income, healthcare card status), health behaviours (smoking, alcohol), and healthcare access (difficulty paying for dental treatment, remoteness). This matching process created comparable groups for analysing the causal pathways using the *MatchIt* package (Ho et al., 2018). A 1:8 ratio was selected to optimise covariate balance and statistical power in our mediation analysis, enhancing comparability between groups. The literature supports higher matching ratios in propensity score methods, particularly in studies with limited sample sizes (Austin, 2011). Before analysis, we verified covariate balance by examining standardised mean differences and visualising distributions (**Supplementary Figure S4**). We then applied the *SparseMCMM* package (Wang et al., 2020) with the matched data, using level of education and income as the treatment variable, dental caries presence/absence as the outcome, and oral microbiome composition (filtered at a genus level using 0.1% abundance and 5% prevalence thresholds) as the mediator. 100 random data splits were incorporated to enhance robustness, and this was used to calculate the direct effects, indirect effects, and total effects. A component-wise effect of each taxon was calculated as a mediator.

## 3 Results

### 3.1 Study population characteristics

The mean age was 41.92 years (SD = 13.37), with mainly female participants (63%) and 91.0% identified as Indigenous (**Supplementary Table S3**). The age distribution showed that nearly half of the participants were in the 35–54 years age group (47.0%), followed by the 18–34 years (34.0%) and >55 years (19.0%) groups. Data collection occurred almost equally across settings, with 55.6% at home/community centres. 58.0% of participants had secondary education or less, while 42.0% had tertiary education. Regarding income sources, 61.0% reported employment as their primary source, while 39.0% received Centrelink payments. HCC possession was evenly distributed (49.0% had cards, 51.0% did not). Financial hardship was prevalent, with 76.0% of participants reporting difficulty paying $100 for dental treatment. Most participants resided in metropolitan areas (86.0%). According to the SEIFA Index, 59.0% lived in low-SES areas, while 41.0% lived in high-SES areas. Access to dental care was limited, with 66.0% reporting their last dental visit was over a year ago. Most (79.8%) visited dentists for problems rather than preventive check-ups (20.2%). Regarding health behaviours, 21% self-reported diabetes, and smoking status was distributed among current smokers (36.0%), non-smokers (38.0%), and past smokers (26.0%). These findings highlight significant socioeconomic disadvantage and barriers to dental care access within this population.

### 3.2 Oral sample type influences oral microbiome diversity and composition

**Figure 1:**
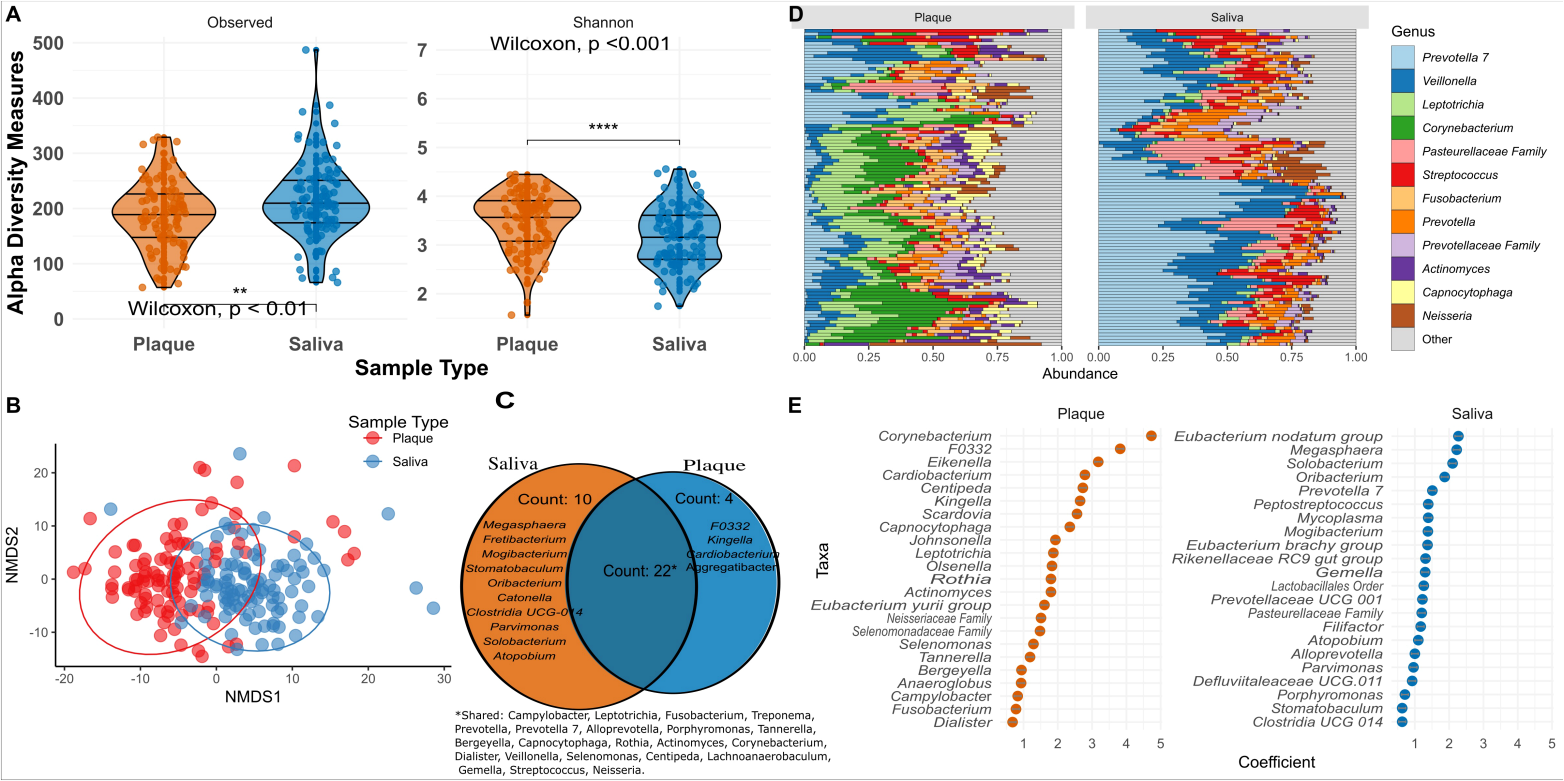
**(A)** Violin plots comparing alpha diversity measures (Observed features and Shannon diversity) between plaque (orange) and saliva (blue) samples. Saliva samples exhibit significantly higher Observed (Wilcoxon p < 0.01) and Shannon diversity (Wilcoxon p <0.001) compared to plaque samples. **(B)** The NMDS plot illustrates that plaque samples (red points) form a distinct cluster, while saliva samples (blue points) are grouped separately. **(C)** Venn diagram showing the core microbiome and unique microbiome in saliva and plaque samples. **(D)** Relative abundance plot as stacked horizontal bars faceted by sample type (plaque and saliva). Genera category labelled “Other” was included to represent less abundant taxa. **(E)** Differential abundance plot showing taxa among saliva and plaque samples.

Saliva and plaque harboured distinct oral microbiomes. Saliva showed consistently higher alpha diversity (Observed features and Shannon index) than plaque (both p < 0.01), and beta diversity analyses demonstrated clear clustering by sample type, confirming that salivary and plaque communities form separate ecological niches (**Figures 1A, B**). A Venn diagram (**Figure 1C**) comparing core microbiome genera between plaque and saliva showed that 22 core genera were shared between the two sample types, including prominent taxa such as *Campylobacter*, *Leptotrichia*, *Fusobacterium*, *Treponema*, *Prevotella*, and *Corynebacterium*. Saliva contained 10 unique core genera, including *Megasphaera*, *Fretibacterium*, *Mogibacterium*, and *Stomatobaculum*, and plaque samples had four unique core genera, which included *F0332*, *Kingella*, *Cardiobacterium* and *Aggregatibacter.* These shared genera represent key members of the oral microbiome that thrive across different niches but may exhibit functional specialisation depending on the environment. The microbial community composition bar plot revealed clear differences between the sample types (**Figure 1D**). Plaque samples were dominated by genera such as *Leptotrichia*, *Corynebacterium*, *Actinomyces*, *Fusobacterium*, and *Capnocytophaga*, which are characteristic of the localised biofilm environment of dental plaque.

In contrast, saliva samples showed higher relative abundances of *Palleniella*, *Veillonella*, *Pasteurellaceae* family, and *Streptococcus*. Differential abundance analysis (**Figure 1E**) further emphasised the distinct microbial profiles with plaque associated with *Corynebacterium*, *F0332*, *Eikenella*, *Cardiobacterium*, and *Centipeda* and saliva associated with genera such as *Megasphaera*, *Solobacterium*, *Oribacterium*, *Palleniella*, and *Peptostreptococcus.* Plaque supports biofilm-associated microbes that are adapted to a localised anaerobic environment, while saliva hosts a broader range of microbial taxa influenced by interactions with external factors.

### 3.3 The salivary and plaque oral microbiomes are uniquely shaped by the sociobiome

**Figure 2:**
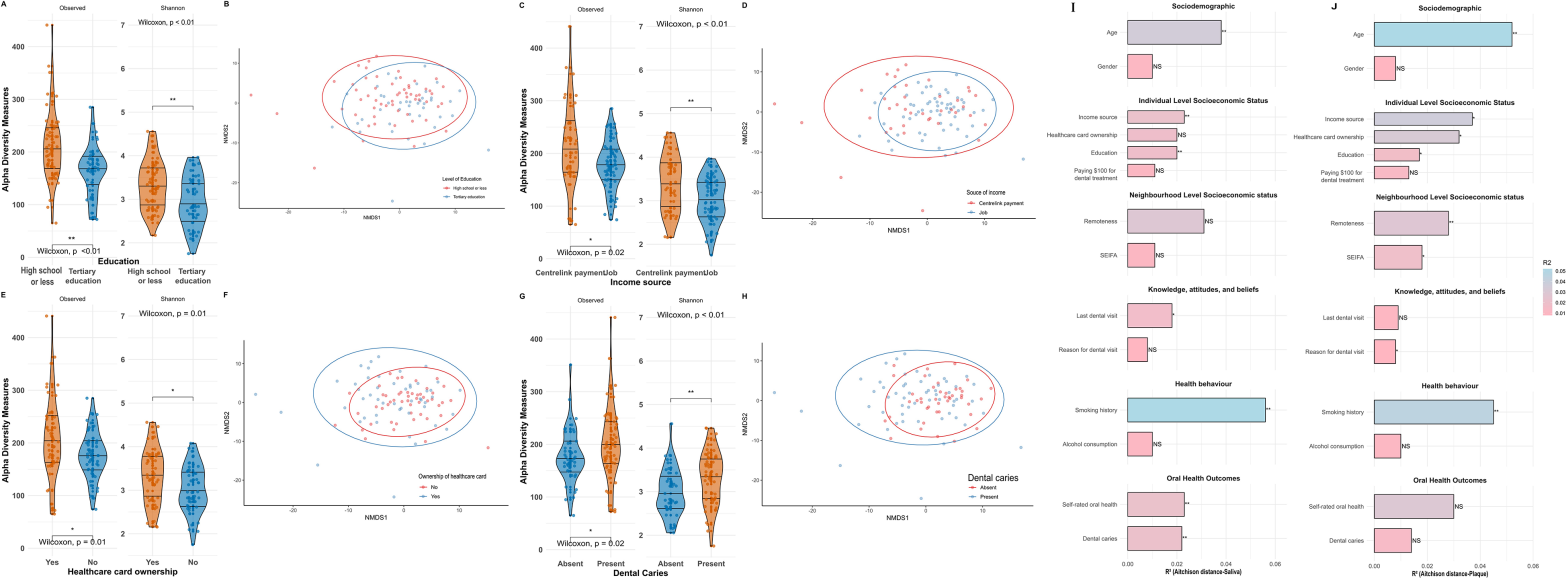
Violin plots of saliva samples showing alpha diversity measures (Observed features and Shannon index) of the salivary samples and NMDS plots based on Aitchison distances visualising beta diversity of the salivary microbiome by **(A, B)** education level (< high school *vs*. tertiary), **(C, D)** income source (Centrelink payment *vs*. job), **(E, F)** Healthcare card ownership (yes *vs*. no), and **(G, H)** dental caries status (present *vs*. absent). Each violin plot compares group distributions, with Wilcoxon *p-values* indicating statistical significance. For NMDS plots, the ellipses represent 95% confidence intervals for each group. **(I, J)** Bar plots summarising the proportion of variance (R²) **(I)** in salivary and **(J)** plaque microbiome composition explained by sociodemographic, individual and neighbourhood-level socioeconomic status, knowledge/attitudes, health behaviours, and oral health outcomes. Bars are coloured by R² value, with significant associations (p <0.05) highlighted.

Socioeconomic disadvantage and behavioural factors were strongly associated with salivary microbiome diversity. Lower education, Centrelink income, HCC ownership, and dental caries were each associated with significantly lower salivary alpha diversity, whereas age-related patterns showed a decline in observed richness with advancing age (**Figures 2A–H**; **Supplementary Table S4**). In the plaque, similar trends were evident but generally weaker, particularly for SES indicators.

The PERMANOVA using Aitchison distances revealed distinct microbial community structures in saliva (**Figure 2I**) and plaque samples (**Figure 2J**) associated with the sociobiome. In the saliva microbiome (**Supplementary Table S5**), the composition was significantly associated with age (R²=0.038, p<0.01), education (R²=0.020, p<0.01), income source (R²=0.038, p<0.01), last dental visit (R²=0.018, p=0.02), smoking history (R²=0.056, p<0.01), self-rated oral health (R²=0.023, p<0.01), and dental caries (R²=0.022, p<0.01). Similarly, PERMANOVA (**Supplementary Table S5**) for plaque samples were significantly associated with age (F = 2.69, R²=0.052, p<0.01), education (R²=0.017, p=0.04), income source (R²=0.037, p=0.01), remoteness (R²=0.018, p=0.02), smoking history (R²=0.045, p<0.01), and self-rated oral health (R²=0.03, p<0.01). Dental caries showed no association with plaque microbiome (p=0.09). These findings suggest that socioeconomic factors (education, income) and behavioural factors (smoking) consistently shape oral microbial communities across different oral habitats, whereas clinical manifestations such as dental caries may have site-specific microbial signatures.

### 3.4 Socioeconomic disadvantage and regional barriers drive dental caries risk

**Figure 3:**
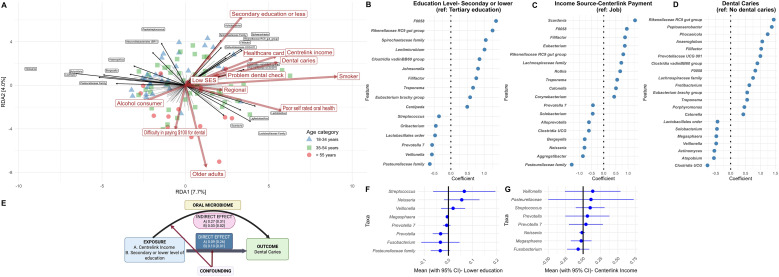
**(A)** Redundancy analysis (RDA) biplot of salivary microbiome composition showing associations with socioeconomic, behavioural, and clinical variables. Arrows represent explanatory variables, with direction and length indicating the strength and direction of association. Sample points are coloured by age category (green triangles: 18–34 years; blue squares: 35–54 years; red circles: >55 years). **(B–D)** Differential abundance analysis of salivary bacterial genera associated with **(B)** education level (secondary or lower *vs*. tertiary education), **(C)** income source (Centrelink payment *vs*. job), and **(D)** dental caries status (present *vs*. absent). Each plot displays the effect size (coefficient) for each taxon, with positive values indicating higher abundance and negative values indicating lower abundance relative to the reference group. **(E)** Causal mediation model illustrating the hypothesised pathway from socioeconomic exposure (Centrelink income or lower education) to dental caries outcome, mediated by oral microbiome composition. The model quantifies the direct effect (blue), the indirect microbiome-mediated effect (pink), and the role of confounding factors. **(F, G)** Forest plots showing the mean and 95% confidence intervals for the mediation effects of key bacterial taxa in the relationship between **(F)** lower education and dental caries, and **(G)** Centrelink income and dental caries. Positive values indicate taxa that mediate increased caries risk in disadvantaged groups; negative values indicate protective mediation.

The RDA (**Figure 3A**) plot for saliva samples demonstrates that dental caries clusters closely with multiple markers of socioeconomic disadvantage, including Centrelink income, HCC ownership, regional residence, and low socioeconomic status. The clustering suggests these variables are strongly intercorrelated and collectively influence microbiome composition in similar directions. Problem-based dental visits align with the low-SES cluster, reinforcing the link between reactive (rather than preventive) dental care utilisation and socioeconomic disadvantage. Age-related patterns were evident, with older adults clustered at the bottom. Overall, dental caries clustered with these socioeconomic disadvantage markers, and individuals with lower SES are more likely to seek dental care only when problems arise.

A differential abundance analysis (**Figures 3B–D**) for saliva samples was plotted for education level (**Supplementary Table S6**), income source (**Supplementary Table S7**) and dental caries (**Supplementary Table S8**). These forest plots illustrate how specific social determinants (education, income source) and clinical outcomes (dental caries) are significantly linked to variations in the abundance of multiple oral microbial taxa. The analysis revealed individuals with secondary education and those receiving Centrelink payments share several significantly abundant taxa that are also elevated in dental caries patients, particularly *Rikenellaceae RC9 gut group* (coefficients =1.05, 0.80, and 1.44 across education, income, and caries), *F0058* (coefficients = 1.36, 0.95, and 0.83), *Filifactor* (coefficients = 0.58, 0.88, and 1.02), and *Treponema* (coefficients = 1.01, 0.55, and 0.55). The findings support a mechanistic relationship between social factors, dental caries and the composition of the salivary microbiome.

### 3.5 Lower income mediates microbiome composition, increasing dental caries risk.

In mediation analysis, the direct effect (DE) of income on dental caries was 0.09 (SE = 0.26), while the mediation effect (ME) operating through the oral saliva microbiome was 0.28 (SE = 0.32). This suggests that approximately 75.6% (ME/TE = 0.28/0.37) of the income-caries relationship operated through changes in the oral microbiome composition (**Figure 3E**; **Supplementary Table S9**). This suggests that lower income may lead to conditions that alter oral bacterial communities, promoting caries development. For education, the direct effect on dental caries remained statistically significant after accounting for the microbiome (DE = 0.10, SE = 0.01; 95% CI 0.08–0.12), while the indirect microbiome-mediated effect was smaller (ME = 0.03, SE = 0.02; 95% CI −0.01–0.07), yielding a total effect of 0.14 (SE = 0.03) (**Supplementary Table S9**). These findings suggest roughly 21% (ME/TE=0.03/0.14) of this disparity was attributable to education-associated differences in oral microbiome composition, while 79% operated through direct pathways. The narrow confidence interval for the direct effect reflects the limited variability remaining after matching and model scaling, but causal interpretation remains constrained by the cross-sectional design and sample size.

Certain microbial taxa play varying roles in mediating the relationship between secondary levels of education and dental caries. (**Figure 3F**; **Supplementary Table S10**). *Streptococcus, Neisseria*, and *Veillonella* demonstrated the strongest positive mediating effects, suggesting that these genera significantly contributed to the pathway through which lower education levels increase caries risk. Conversely, *Fusobacterium*, *Prevotella*, and *Pasteurellaceae* displayed a negative mediating effect (wide standard error suggests this estimate should be interpreted cautiously). Among participants receiving Centrelink income*, Veillonella*, *Pasteurellaceae*, *Streptococcus*, and *Prevotella* were more abundant, and these genera are linked to increased caries risk (**Figure 3G**, **Supplementary Table S11**). Given this imprecision, these mediators are considered exploratory and hypothesis-generating rather than definitive findings.

## 4 Discussion

This study provides the first evidence that socioeconomic disadvantage in Indigenous Australian adults is biologically embedded in the oral microbiome and that these microbial changes substantially mediate the relationship between income and dental caries. Notably, about 75% of the link between lower income (Centrelink dependence) and dental caries risk occurs via changes in the oral microbiome. These findings have significant implications for understanding the biological mechanisms underlying persistent oral health disparities experienced by Indigenous communities, moving beyond behavioural explanations to recognise how structural disadvantage becomes biologically embedded through changes in the oral microbiome. Dietary patterns shaped by food insecurity, particularly frequent consumption of sugar-rich, nutrient-poor foods, select for acidogenic and aciduric microbial communities, thereby increasing the cariogenic potential of the oral biofilm (e.g., *Streptococcus mutans*, *Lactobacillus*) (Nath et al., 2025). Chronic stress and adverse life experiences, prevalent among disadvantaged groups, are associated with altered salivary flow, elevated cortisol levels, and dysregulation of host immune responses, thereby facilitating pronounced shifts in oral microbiome structure (Handsley-Davis et al., 2021).

A key finding from our study is the differential impact of sampling methods on capturing socioeconomic associations. While plaque and saliva samples showed significant associations with age, education, income source, and smoking history, saliva microbiome composition uniquely demonstrated significant relationships with healthcare card status, patterns of last dental visits, and dental caries presence. This indicates that saliva could be a better medium for exploring socioeconomic effects on oral microbiota and related health issues (Shi et al., 2018). This observation aligns with work with Indigenous children, where salivary characteristics were significantly associated with microbiota diversity and composition (Handsley-Davis et al., 2022). The greater sensitivity of salivary samples to socioeconomic gradients may reflect saliva’s role as an integrative fluid representing the entire oral cavity rather than site-specific plaque biofilms, providing a more comprehensive view of the oral ecosystem shaped by various socioeconomic exposures over time (Kageyama et al., 2017; Shi et al., 2018).

The RDA analysis further emphasised how socioeconomic disadvantage shapes salivary microbiome composition, with education levels emerging as the strongest determinant, followed closely by income source and HCC status. This convergence indicates that salivary microbiome signatures could be used to identify adults at heightened caries risk linked to social disadvantage and may guide targeted preventive strategies, such as intensified recall schedules or microbiome-modulating interventions, in community and primary care settings. The differential abundance analysis provided further evidence for this pathway, identifying specific bacterial taxa (*Rikenellaceae RC9 gut group*, *F0058*, *Filifactor*, and *Treponema*) that were consistently associated in both low SES environments and individuals with dental caries. These patterns align with studies of Indigenous Australian children, where higher microbial diversity was linked to proxies for lower socioeconomic status and less frequent toothbrushing (Handsley-Davis et al., 2022).

Mediation analysis indicated that around three-quarters of the association between lower income (Centrelink dependence) and dental caries operated through alterations in salivary microbiome composition. Despite having access to public dental care, high caries rates persist due to systemic gaps in preventive care, financial barriers, and underlying socioeconomic determinants. Public dental services often prioritise emergency treatments over preventive care, with waitlists exceeding 12–24 months in many regions (Dudko et al., 2016). This gap between theoretical access and practical utilisation mirrors findings from previous Australian studies demonstrating that Indigenous Australians disproportionately experience poor oral health, with limited access to culturally appropriate and timely dental care being a significant factor (Jamieson et al., 2023).

Our study has several methodological strengths. (1) Including both plaque and saliva samples enabled direct comparison of these sampling methods for detecting socioeconomic gradients. (2) The application of mediation analysis allowed us to quantify the contribution of microbiome alterations to the overall relationship between SES and dental caries. (3) Focusing on Indigenous Australians addresses an important gap in the literature, as most oral microbiome research has been conducted in non-Indigenous populations despite evidence that Indigenous communities may have unique oral microbiota shaped by distinct genetic, cultural, and environmental factors (Skelly et al., 2018; Nath et al., 2021a). Several limitations warrant consideration. First, the use of convenience sampling limits the generalizability of findings to the broader Indigenous Australian population. While our sample captured diverse socioeconomic circumstances, it may not fully represent all Indigenous communities, particularly those in very remote areas. Second, the cross-sectional design precludes causal inference. This constraint is particularly relevant for the mediation analysis, which assumes that socioeconomic exposures precede microbiome changes and that microbiome alterations precede caries onset. Because all variables were measured at a single time point, these temporal sequences cannot be empirically verified, and the estimated direct and indirect effects should therefore be interpreted as indicative pathways rather than definitive causal quantities. Third, we did not measure several potential confounders that may influence oral microbiome composition, including detailed dietary patterns (frequency and type of sugar consumption), oral hygiene behaviours (toothbrushing frequency, fluoride toothpaste use), and genetic factors. A full dietary questionnaire was not included at baseline to minimise respondent burden during home and community−based visits and to prioritise socioeconomic, behavioural and clinical measures within an already extensive mixed−methods protocol. These unmeasured variables may partially explain observed associations. Fourth, variability in sample collection settings (home, community centre, clinic) and storage duration (2–18 months at -20 °C prior to sequencing) may have introduced technical variation. Lastly, the mediation analysis suggests a substantial role for microbiome-mediated effects; the wide standard errors in these models indicate considerable uncertainty in the estimates. The negative mediation estimates should be interpreted cautiously, as they suggest these patterns might result from sampling variability or model instability rather than true protective effects.

The microbiome-mediated effects of income on dental caries risk suggest that interventions targeting microbial ecology, such as water fluoridation, professionally applied topical antimicrobials or prebiotics promoting beneficial bacteria, may help mitigate socioeconomic disparities in oral health outcomes (Nath et al., 2024). However, the concurrent finding that education’s impact operates primarily through non-microbiome pathways underscores the need for multipronged approaches addressing both biological and social determinants of oral health. Future longitudinal or intervention studies that repeatedly sample the microbiome and clinical status will be essential to confirm the directionality and stability of these mediating relationships. Integration of metagenomic shotgun sequencing in future studies will enable targeted investigation of microbial functions that may underlie the SES-caries pathway (Boyce et al., 2010). We recommend prioritising the analysis of metabolic pathways related to carbohydrate fermentation and acidogenesis, genes associated with biofilm formation and maturation, and microbial virulence factors such as adhesins and proteases. Further investigations into antimicrobial resistance determinants and immunomodulatory molecules may clarify how socioeconomic context shapes oral microbiome function and influences host susceptibility to dental caries.

## 5 Conclusion

This study demonstrates that socioeconomic disadvantage significantly shapes oral microbiome composition in Indigenous Australians, with salivary microbiota serving as a sensitive indicator of these social gradients. By applying the sociobiome framework to an Indigenous adult population, this work provides the first empirical evidence of a microbiome-mediated pathway underpinning Indigenous oral health inequities. Addressing these inequities will require integrated strategies that combine upstream policies targeting socioeconomic conditions with downstream, microbiome-informed preventive interventions tailored to Indigenous communities.

## Data Availability

The datasets presented in this study can be found in online repositories. The names of the repository/repositories and accession number(s) can be found in the article/**Supplementary Material**.
