# Supplementary material for: The sociobiome–oral microbiome mediates dental caries among Indigenous Australians
Source: Front Cell Infect Microbiol. 2025 Dec 17;15:1721183. doi: 10.3389/fcimb.2025.1721183 (PMC12753951; doi:10.3389/fcimb.2025.1721183)
Supplement: Supplementary file 1 [file Table1.docx]

Table of Contents

[SUPPLEMENTARY TEXT S1. VARIABLES 2](#_Toc215166410)

[SUPPLEMENTARY TEXT S2. SAMPLE COLLECTION 4](#_Toc215166411)

[SUPPLEMENTARY TEXT S3. DNA EXTRACTION, AMPLIFICATION, AND SEQUENCING 6](#_Toc215166412)

[SUPPLEMENTARY TEXT S4. BIOINFORMATICS ANALYSIS 7](#_Toc215166413)

[4.1. Data pre-processing: 7](#_Toc215166414)

[4.2 Contaminant detection and removal 7](#_Toc215166415)

[4.3 Filtering steps 8](#_Toc215166416)

[FIGURES 10](#_Toc215166417)

[Figure S2. The identification of contaminants in biological samples and in control samples 11](#_Toc215166418)

[Figure S3. Alpha rarefaction plot by sample type 13](#_Toc215166419)

[Figure S4. The love plot displays standardised mean differences before and after matching for the confounding factors. 13](#_Toc215166420)

[SUPPLEMENTARY TABLES 15](#_Toc215166421)

[Table S1. List of contaminants from the negative controls 15](#_Toc215166422)

[Table S2. List of contaminants from the positive controls 18](#_Toc215166423)

[Table S3. Participant characteristics of the analysed cohort (n=100) and overall participants in the study (n=280) 20](#_Toc215166424)

[Table S4. Alpha diversity test results for observed feature metrics and Shannon’s diversity metrics from plaque and saliva samples 22](#_Toc215166425)

[Table S5. Beta diversity using significance test results using Aitchison distances for plaque and saliva samples 24](#_Toc215166426)

[Table S6. Results from differential abundance analysis for the secondary level of education (ref. Tertiary education) 25](#_Toc215166427)

[Table S7. Results from differential abundance analysis for income source as Centrelink (ref. Job) 26](#_Toc215166428)

[Table S8. Results from differential abundance analysis for the presence of dental caries (ref. no caries) 27](#_Toc215166429)

[Table S9. Results from the mediation analysis of income source and education as the exposure and dental caries as the outcome. 28](#_Toc215166430)

[Table S10. Component-wise mediation effect of individual microbial taxa on the relationship between secondary education and dental caries. 28](#_Toc215166431)

[Table S11. Component-wise mediation effect of individual microbial taxa on the relationship between Centrelink income and dental caries. 28](#_Toc215166432)

## SUPPLEMENTARY TEXT S1. VARIABLES

Domains of social determinants of health.

| **Domain** | **Variables** |
| --- | --- |
| Sociodemographic | Age, Sex |
| Socioeconomic | Individual level- Education, Income source, ownership of healthcare card, dental affordability.  Neighbourhood level- Remoteness, The Socio-Economic Indexes for Areas |
| Knowledge, attitudes, and beliefs | Last dental visit, reason for last dental visit. |
| Health behaviour variables | Self-reported diabetes, smoking, alcohol consumption |
| Oral health factors | Self-reported oral health, dental caries. |

Age was categorised into “18-34 years”, “35-54 years”, and “≥ 55 years”. Gender was classified as ‘Male’ or ‘Female’.

Education was based on the highest level of school completed, categorised as ‘High school’ or less, and ‘University’ and “Trade or TAFE” classified under tertiary education. TAFE stands for ‘Technical and Further Education’ and provides training for vocational occupations. The main sources of income were dichotomised as “Centrelink payments” (welfare support payments by the Australian government) or “Job” or “others” when not meeting any of the above criteria. The ownership of a government-administered health care card (HCC) was recorded as “Yes” vs “No”; an HCC is means-tested and enables access to services such as publicly funded dental care. For dental affordability, the participants were asked if they had “difficulty paying a $100 dental bill” and was categorised into two groups, "Hard," included responses such as "could not pay," "very hard," and "a little bit hard," and as "Not hard," which included responses "not hard at all" and “not very hard”.

Remoteness was categorised into “Metropolitan” or “Regional/Remote” based on the Australian Bureau of Statistics – Australian Statistical Geographical Standard derived from the postcode of selected individuals [1]. The Socio-Economic Indexes for Areas (SEIFA) is a set of four indexes developed by the Australian Bureau of Statistics (ABS) to measure socioeconomic status at the area level [2]. These indices include the Index of Relative Socio-economic Disadvantage (IRSD), the Index of Relative Socio-economic Advantage and Disadvantage (IRSAD), the Index of Economic Resources (IER), and the Index of Education and Occupation (IEO). For this study, the IRSD was employed as the area-based composite measure of socioeconomic status. The IRSD provides a summary of the economic and social conditions within an area, with a focus on relative disadvantage. It incorporates various factors such as income, education, unemployment, and occupational skills. The IRSD score of each participant's residential Statistical Area Level 1 (SA1-maximum score) was utilised as a measure of socioeconomic status. Lower IRSD scores indicate a relatively greater disadvantage, while higher scores suggest a relative lack of disadvantage in the area.

Self-reported diabetes was dichotomised into ‘Diabetes’ or ‘No diabetes’. The assessment of smoking habits was categorised into three distinct groups; current smokers were identified by "yes" response, past smoking history was recorded based on the response "I don't smoke now, but I used to," and non-smokers as those who responded that they had "never smoked in a lifetime". Alcohol consumption was evaluated using a two-tiered approach. Initially, participants were asked if they consumed alcohol, with responses recorded as either "Yes" or "No." For those who answered “Yes”, the frequency of alcohol consumption was further classified into three categories: "weekly" or "monthly".

Oral health variables included self-rated oral health (SROH) and dental visiting behaviours. SROH included “good” and “poor”. To determine the recency of dental care, participants were asked, "How long ago did you last see a dental professional about your teeth, dentures or gums?" Responses were categorised into two groups: those who had visited within the past year and those whose last visit was a year ago or more. The reason behind dental visits was also examined, with responses dichotomised into either 'check-up' or 'problem' as the primary reason.

## SUPPLEMENTARY TEXT S2. SAMPLE COLLECTION

Strict aseptic techniques were maintained throughout the procedure, with the dental professional wearing a mask, gloves, and protective eyewear. Each participant was provided with a collection tube (Zymo DNA/RNA Shield SafeCollect Saliva Collection Kit) and asked to sit comfortably and upright for saliva collection. Participants were instructed to allow saliva to pool in their mouth for about a minute before expectorating directly into the tube. This process was repeated for approximately 15 minutes or until 2 mL of saliva was collected. Once the collection was complete, the Zymo buffer solution was added to the tube, and the tube was securely capped and gently inverted 10-15 times to mix the saliva with the buffer solution.

For supragingival plaque collection, six sites were selected: the mesio-buccal surfaces of a right maxillary molar, a left mandibular molar, a right mandibular molar, and a mesio-palatal surface of a maxillary using a Gracey curette [3]. Index teeth were isolated with cotton rolls and gently dried with air. The collected plaque was transferred into a 2ml Sube containing Zymo DNA/RNA shield. The curette tip was immersed in the solution for 5-10 seconds with slight shaking, and its surface was wiped on the inside edge of the tube to ensure complete transfer. This process was repeated for all selected teeth. A trained dental professional used a sterile curette to carefully remove visible calculus deposits from the surfaces of the teeth, focusing on areas of heaviest accumulation, typically the lingual surfaces of the lower anterior teeth and the buccal surfaces of the upper molars. The collected calculus was immediately transferred into a 2ml tube containing a Zymo DNA/RNA shield. Care was taken to ensure minimal disruption of the soft tissue to avoid bleeding and contamination of the sample. The process was repeated on multiple teeth until a sufficient amount of calculus (approximately 5-10 mg) was collected. Once the collection was complete, each tube was labelled with the participant identifier, researcher initial, collection location and collection date and then stored at -20°C until further analysis.

Environmental control samples were collected to account for potential airborne contamination during the sampling process, which included environmental and curette washes. For each sampling session, a 2ml collection tube containing a DNA/RNA shield (Zymo Research, California, USA) (identical to those used for oral samples) were labelled with an appropriate environmental control sample ID. The tube was then opened and placed in a tube rack at a representative location within the sampling area, ensuring it was not directly under any vents or fans. The open tube was left exposed to the air for the duration of data collection. After the exposure period, the tube was carefully capped without touching the inside of the tube or cap. The sample ID, date, time, and location are recorded on a data collection sheet. The collection of curette wash samples was an additional environmental control measure in the study protocol. This process assessed potential contamination from the dental instruments used during sample collection. A sterile curette was swirled thoroughly in a 2 ml collection tube containing a DNA/RNA shield. After collection, both the control samples were stored and processed in the same manner as the oral microbiome samples.

## SUPPLEMENTARY TEXT S3. DNA EXTRACTION, AMPLIFICATION, AND SEQUENCING

DNA was extracted in a dedicated pre-PCR laboratory at the microARCH laboratory at Pennsylvania State University using the ZymoBIOMICS DNA miniprep kit, following the manufacturer's instructions. The RIDE checklist [4]was followed, which involved reporting methodology, including negative controls, determining contamination levels, and exploring contamination downstream. For each batch of DNA extractions (n = 12), an extraction blank control (EBC) was included, which was also processed in all subsequent analyses to assess contamination. The V4 region of the 16S rRNA gene was targeted using the uniquely barcoded reverse primer for each sample [5]. Alongside each amplification, the no-template controls (NTC) were processed. The amplified, barcoded DNA was quantified using the Invitrogen Qubit dsDNA BR assay (Life Technologies), pooled at equal relative concentrations, purified with 1.1X Axygen AxyPrep Mag™ PCR Clean-up beads, and quantified using the D1000 reagents on the TapeStation (Agilent, Santa Clara, CA, USA). Paired-end 150bp sequencing was performed using an IlluminaMiSeq 2300 platform (Illumina, Inc. San Diego) at the Pennsylvania State University Genomics Core Facility.

## SUPPLEMENTARY TEXT S4. BIOINFORMATICS ANALYSIS

4.1. Data pre-processing: The microbiome data was processed using Quantitative Insights Into Microbial Ecology 2 (QIIME2 version 2024.11), starting with importing FASTQ files [6]. The forward and reverse reads were merged into a single file. Initial quality checks were performed using the *qiime demux summarize* function to assess the raw data. Denoising was conducted with the *q2-deblur plugin* to enhance sequence quality. The sequences were trimmed to 200 base pairs for forward and reverse reads. Representative sequences were generated using the *q2-feature-table-tabulate-seqs* command. A phylogenetic tree was constructed using the *phylogeny align to tree mafft fasttree* command, which is essential for downstream analyses. Amplicon sequence variants (ASVs) were created, and taxonomic classifications were determined using the Silva 138 database (99% full-length sequences) with the *feature classifier classify sklearn* command. This comprehensive workflow ensured high-quality data for further analysis.

### 4.2 Contaminant detection and removal

The next step was to remove possible contaminants from the biological samples. A three-pronged approach to environmental control sampling (air exposure, curette wash, and standard negative controls) was used to distinguish between airborne contaminants, instrument-related contaminants, and any systematic contamination in the sampling process. In RStudio version 2024.09.0, the packages *decontam* [7], *phyloseq* [8], and *qiime2R* [9] were used to detect and remove microbes in the controls. We used four types of controls to monitor contamination in our study: EBCs (extraction blank control), NTCs (no-template controls), and environmental controls (ENV) curette wash (CW). These controls are crucial to mitigate environmental and laboratory-based contamination that can distort microbial studies [4].

The contaminants were removed in two steps, as cross-contamination can lead to false-positive results. In the first step, the contaminant profiles within EBC and NTC-negative control samples were compared to biological samples in the study to mitigate contamination introduced through the sample processing. There was a significant difference in diversity and composition between the samples and the controls (Figure S1A). First, the prevalence of features from EBC and NTC (Figure S1B) was considered, and 54 contaminant taxa were detected and removed (Table S1). Next, contaminants from the ENV and CW were assessed (Figure S1C) and removed to account for contamination introduced during sample collection, including 39 contaminant taxa (Table S2). These contaminant analyses were done separately to account for the spill-over effect from the higher microbial biomass samples, such as the environment and the dental curettes [4].

### 4.3 Filtering steps

After both decontamination steps, the microbiota counts, sample metadata and taxonomy artefacts were imported into RStudio to create a phyloseq object using the *phyloseq package* [8] for further analysis. The initial processing included checking for ASV distribution, depth of sample reads, and missing ASVs.

The data filtering was conducted in four steps to refine the microbiome dataset: removing ASVs with zero counts, removing rare and low abundance ASVs, and removing contaminants. Initially, amplicon sequence variants (ASVs) with zero counts across all samples were removed. This step ensured that only meaningful data was retained for further analysis. Following this, a prevalence filter was applied to retain only ASVs in more than 5% of the samples. This step helped eliminate rare or spurious taxa that might not contribute significantly to the microbial community structure. Subsequently, an abundance threshold filter was applied, removing ASVs with a total abundance of fewer than 10 reads across all samples. This ensured that only taxa with a substantial presence were considered in the analysis. Contaminants, specifically chloroplasts and mitochondria, were removed to focus on microbial taxa of interest. Finally, the samples were sorted by read depth.

References

1. Australian Bureau of Statistics (Jul2021-Jun2026). Remoteness Areas, ABS. <https://www.abs.gov.au/statistics/standards/australian-statistical-geography-standard-asgs-edition-3/jul2021-jun2026/remoteness-structure/remoteness-areas>. Accessed 26 February 2025.

2. Australian Bureau of Statistics: Census basic community profile and snapshot. In.: Australian Bureau of Statistics Adelaide, SA, Australia; 2001.

3. Nath S, Zilm P, Jamieson L, Kapellas K, Goswami N, Ketagoda K, et al. Development and characterization of an oral microbiome transplant among Australians for the treatment of dental caries and periodontal disease: A study protocol. PLoS One. 2021;16(11):e0260433.

4. Eisenhofer R, Minich JJ, Marotz C, Cooper A, Knight R, Weyrich LS. Contamination in low microbial biomass microbiome studies: issues and recommendations. Trends in microbiology. 2019;27(2):105-17.

5. Caporaso JG, Lauber CL, Walters WA, Berg-Lyons D, Huntley J, Fierer N, et al. Ultra-high-throughput microbial community analysis on the Illumina HiSeq and MiSeq platforms. Isme j. 2012;6(8):1621-4; doi: 10.1038/ismej.2012.8.

6. Bolyen E, Rideout JR, Dillon MR, Bokulich NA, Abnet CC, Al-Ghalith GA, et al. Reproducible, interactive, scalable and extensible microbiome data science using QIIME 2. Nature Biotechnology. 2019;37(8):852-7; doi: 10.1038/s41587-019-0209-9.

7. Davis NM, Proctor DM, Holmes SP, Relman DA, Callahan BJ. Simple statistical identification and removal of contaminant sequences in marker-gene and metagenomics data. Microbiome. 2018;6(1):226; doi: 10.1186/s40168-018-0605-2.

8. McMurdie PJ, Holmes S. phyloseq: an R package for reproducible interactive analysis and graphics of microbiome census data. PLoS One. 2013;8(4):e61217; doi: 10.1371/journal.pone.0061217.

9. Bisanz JE. qiime2R: Importing QIIME2 artifacts and associated data into R sessions. Version 099. 2018;13.

## FIGURES

Figure S1. Analysing the contamination in biological samples, and controls and identifying the contaminants in the samples.

1. The Principal Coordinate Analysis (PCoA) plot shows the biological samples (blue dots), controls (red dots). There was a clear separation seen between biological samples and controls. The analysis was conducted in QIIME2.


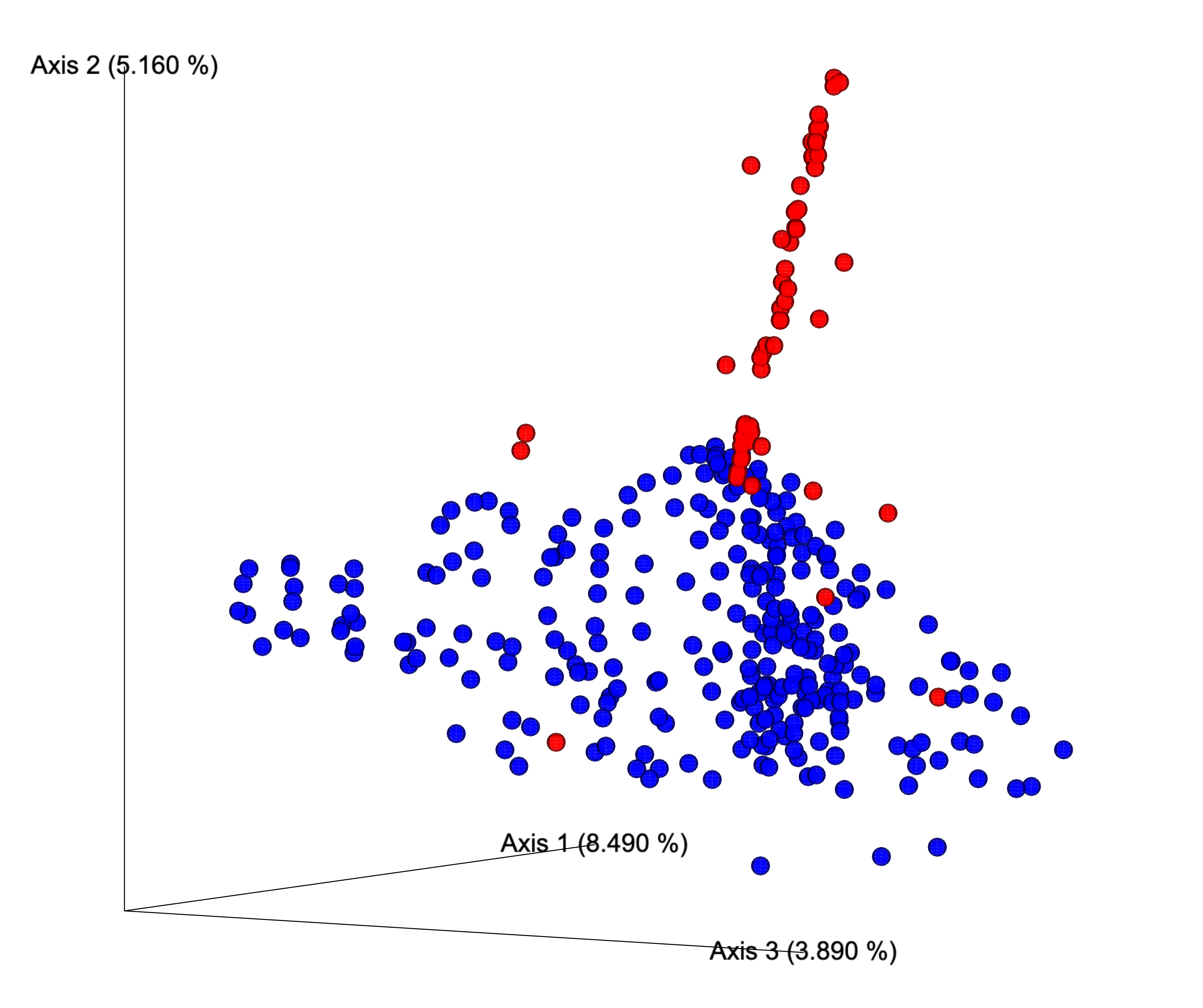


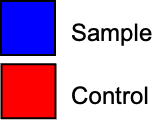


1. The Principal Coordinate Analysis (PCoA) plot shows the biological samples (blue dots), extraction blank controls (EBCs; purple dots) PCR controls (pink dots), environmental control (yellow dots) and curette wash (CW) controls (green dots). There was a clear separation seen between biological samples and controls. The analysis was conducted in QIIME2.


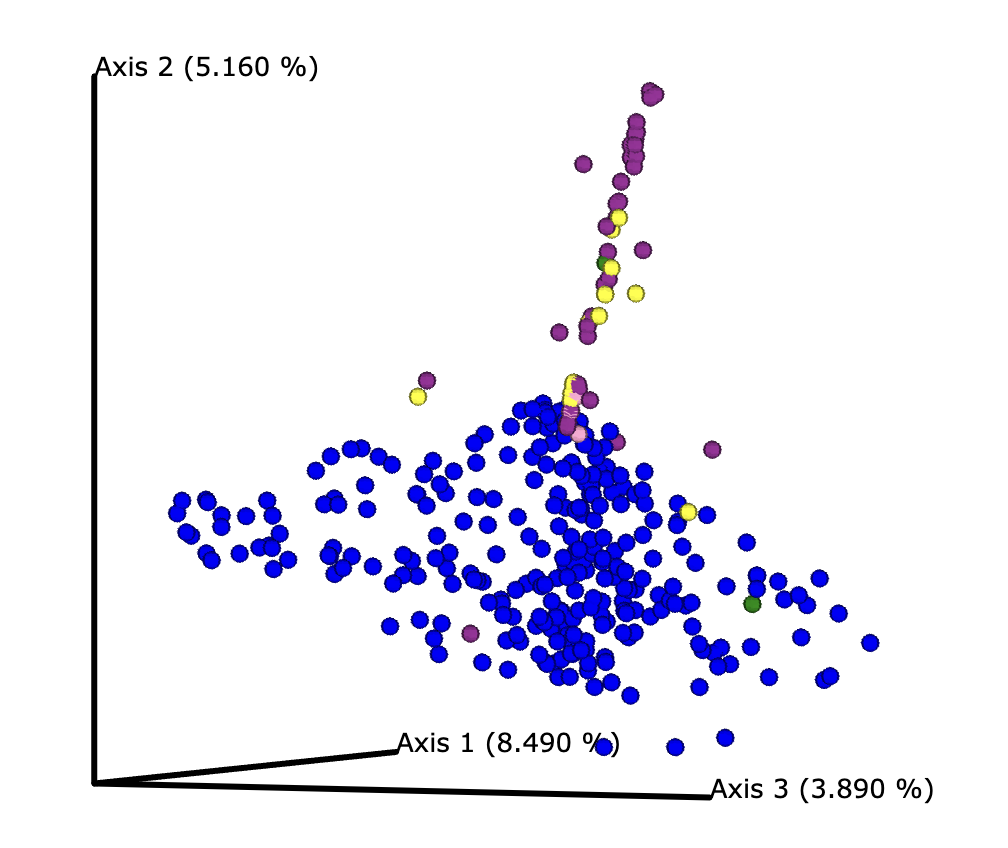


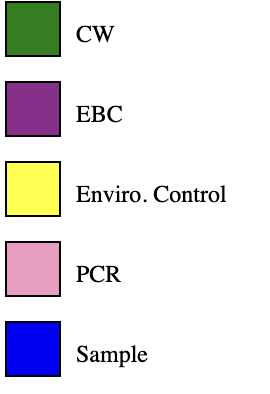


Figure S2. The identification of contaminants in biological samples and in control samples.

The plot displays the results of contaminant identification using the *Decontam* package, which distinguishes true microbial taxa from contaminants based on their prevalence in biological samples versus negative controls (extraction blank controls [EBC] and PCR blanks). The x-axis represents the prevalence of taxa in negative controls, while the y-axis shows their prevalence in true samples. Each point corresponds to a taxon, with its colour indicating whether it was classified as a contaminant (blue, labelled "TRUE") or not (red, labelled "FALSE").

#### A. Identification of contaminants from biological samples and positive controls (Extraction blank controls and no template controls)


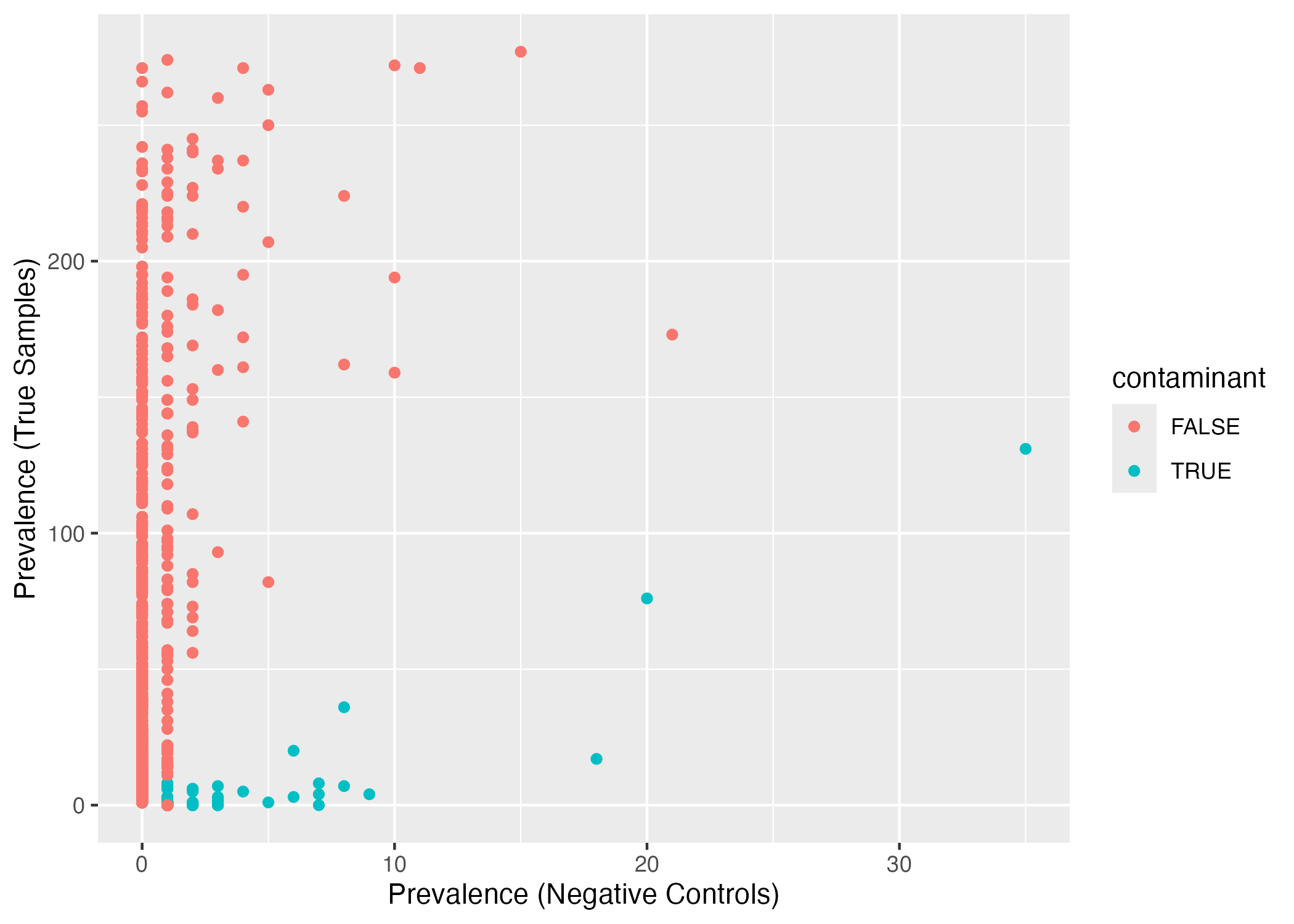


#### Identification of contaminants from biological samples and positive controls (environmental controls and curette wash)


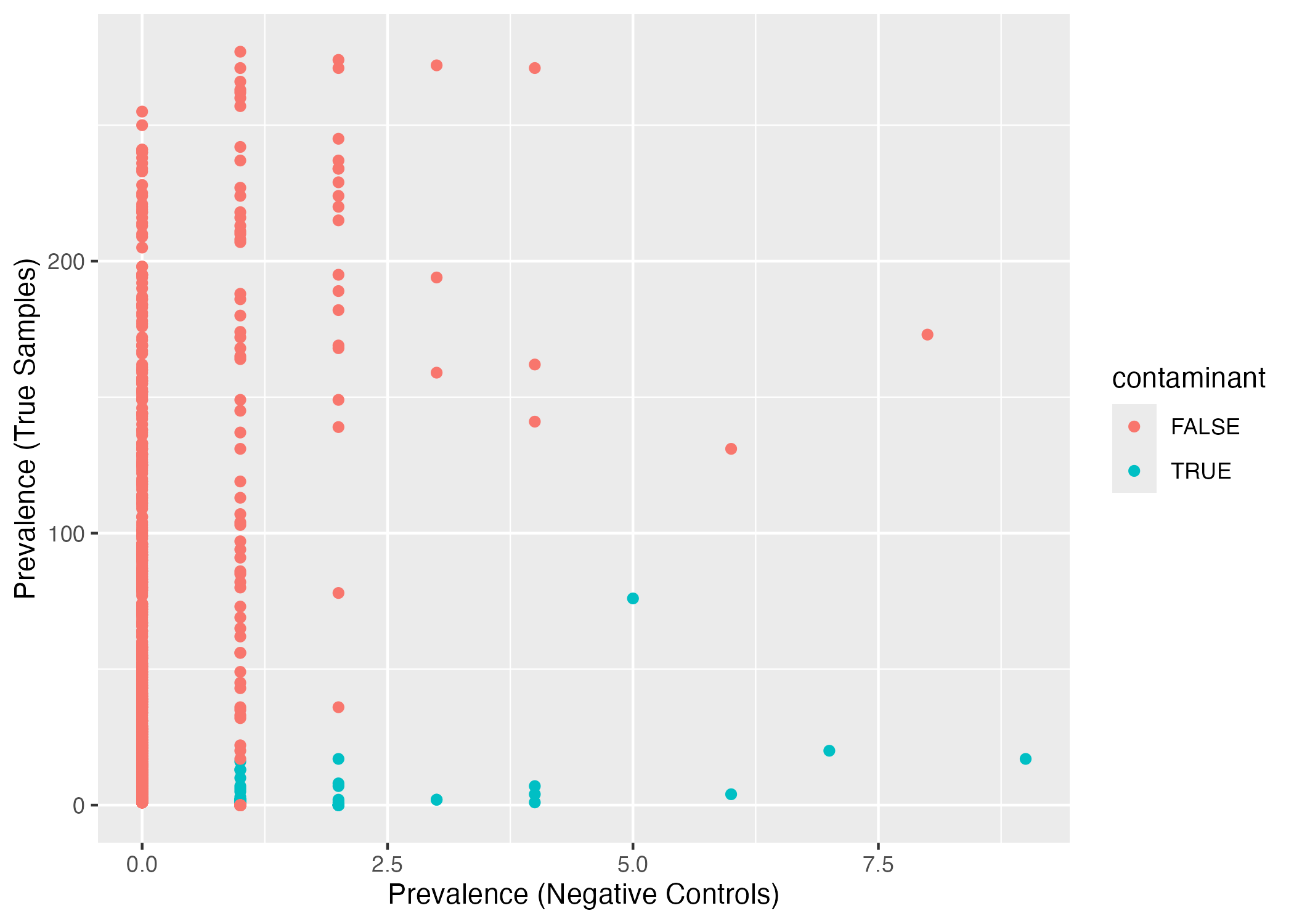


### Figure S3. Alpha rarefaction plot by sample type

Alpha rarefaction curves displaying Shannon diversity as a function of sequencing depth for two different sample types: plaque (red) and saliva (blue). Each line represents an individual sample.
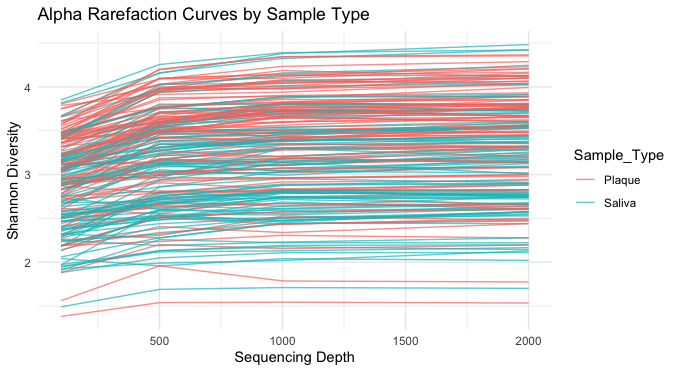


### Figure S4. The love plot displays standardised mean differences before and after matching for the confounding factors.

#### The income variable is the exposure.


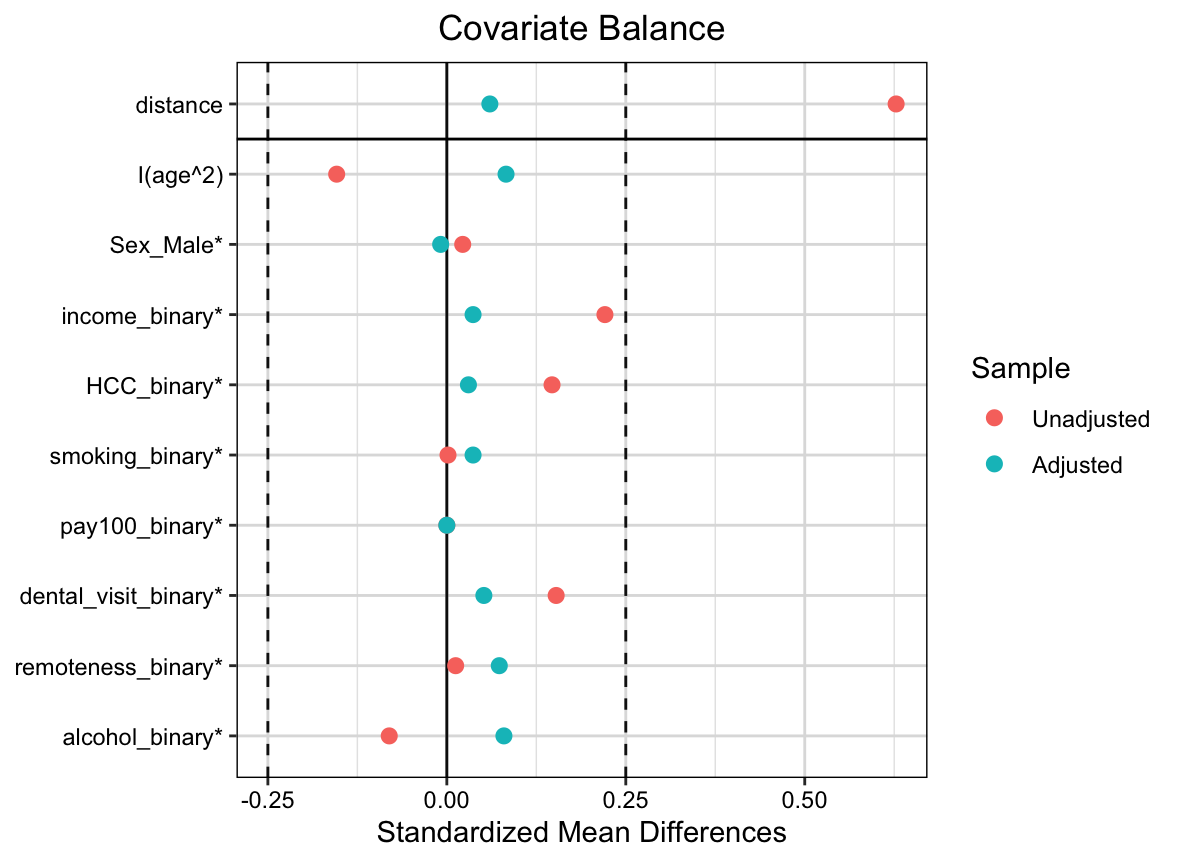


#### The education variable is the exposure.


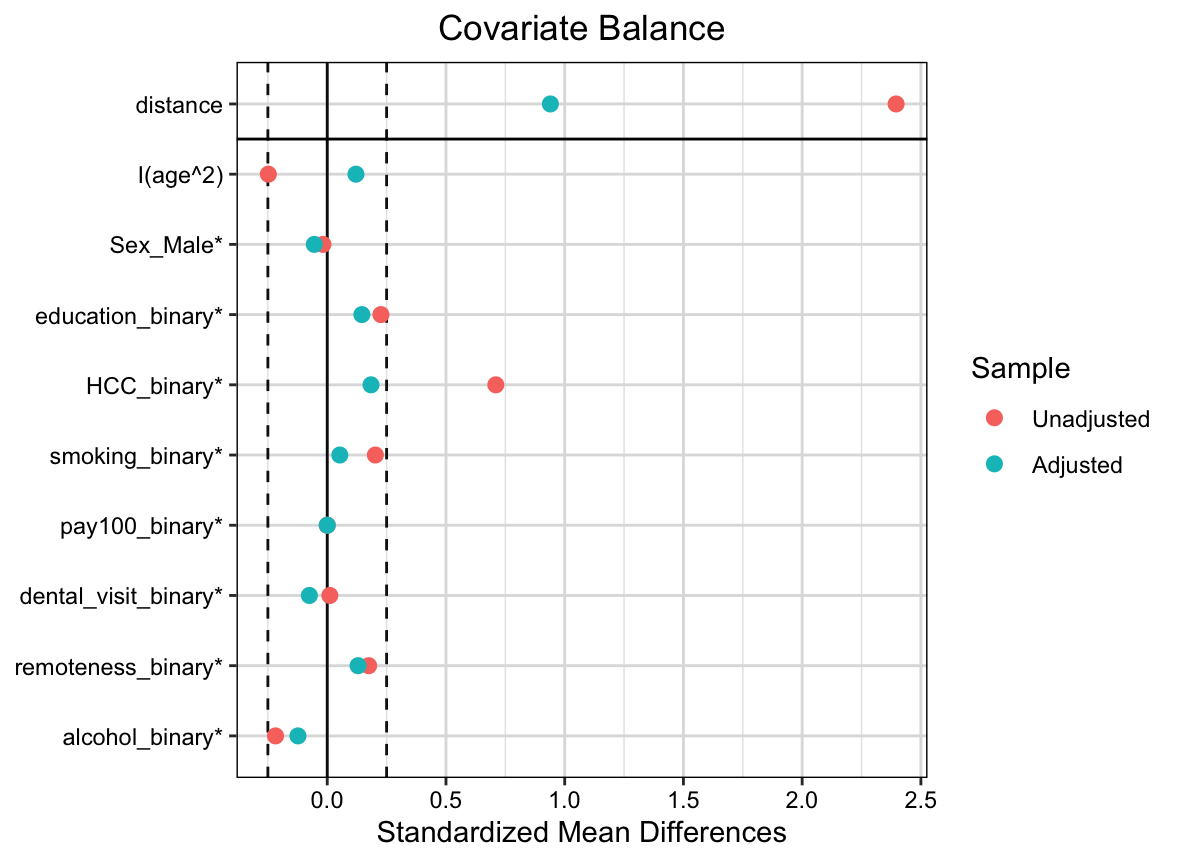


##

## SUPPLEMENTARY TABLES

### Table S1. List of contaminants from the negative controls

The feature ID and feature taxonomy were assigned using the SILVA 132 database. 54 Features were identified as contaminants in *decontam* package. The contaminants were more prevalent in negative controls (Extraction Blank Controls and No Template Controls) than in supragingival plaque samples.

| **Kingdom** | **Phylum** | **Class** | **Order** | **Family** | **Genus** |
| --- | --- | --- | --- | --- | --- |
| d__Bacteria | *Proteobacteria* | *Gammaproteobacteria* | *Burkholderiales* | *Comamonadaceae* | NA |
| d__Bacteria | *Fusobacteriota* | *Fusobacteriia* | *Fusobacteriales* | *Leptotrichiaceae* | *Leptotrichia* |
| d__Bacteria | *Proteobacteria* | *Gammaproteobacteria* | *Pseudomonadales* | *Moraxellaceae* | *Acinetobacter* |
| d__Bacteria | *Proteobacteria* | *Gammaproteobacteria* | *Pseudomonadales* | *Pseudomonadaceae* | NA |
| d__Bacteria | *Proteobacteria* | *Gammaproteobacteria* | *Pseudomonadales* | *Pseudomonadaceae* | *Pseudomonas* |
| d__Bacteria | *Actinobacteriota* | *Actinobacteria* | *Actinomycetales* | *Actinomycetaceae* | *Actinomyces* |
| d__Bacteria | *Bacteroidota* | *Bacteroidia* | *Bacteroidales* | *Prevotellaceae* | *Prevotella_7* |
| d__Bacteria | *Firmicutes* | *Bacilli* | *Lactobacillales* | *Lactobacillaceae* | *Lactobacillus* |
| d__Bacteria | *Proteobacteria* | *Gammaproteobacteria* | *Burkholderiales* | *Neisseriaceae* | *uncultured* |
| d__Bacteria | *Proteobacteria* | *Gammaproteobacteria* | *Pseudomonadales* | *Pseudomonadaceae* | *Pseudomonas* |
| d__Bacteria | *Actinobacteriota* | *Actinobacteria* | *Corynebacteriales* | *Corynebacteriaceae* | *Corynebacterium* |
| d__Bacteria | *Proteobacteria* | *Gammaproteobacteria* | *Pseudomonadales* | *Moraxellaceae* | *Enhydrobacter* |
| d__Bacteria | *Proteobacteria* | *Gammaproteobacteria* | *Burkholderiales* | *Comamonadaceae* | NA |
| d__Bacteria | *Proteobacteria* | *Gammaproteobacteria* | *Pseudomonadales* | *Moraxellaceae* | *Acinetobacter* |
| d__Bacteria | *Proteobacteria* | *Gammaproteobacteria* | *Pseudomonadales* | *Moraxellaceae* | *Acinetobacter* |
| d__Bacteria | *Proteobacteria* | *Gammaproteobacteria* | *Burkholderiales* | *Alcaligenaceae* | *Alcaligenes* |
| d__Bacteria | *Proteobacteria* | *Alphaproteobacteria* | *Caulobacterales* | *Caulobacteraceae* | *Brevundimonas* |
| d__Bacteria | *Proteobacteria* | *Gammaproteobacteria* | *Burkholderiales* | *Oxalobacteraceae* | *Janthinobacterium* |
| d__Bacteria | *Proteobacteria* | *Gammaproteobacteria* | *Burkholderiales* | *Oxalobacteraceae* | *Herminiimonas* |
| d__Bacteria | *Bacteroidota* | *Bacteroidia* | *Bacteroidales* | *Prevotellaceae* | *Prevotella 7* |
| d__Bacteria | *Firmicutes* | *Clostridia* | *Peptostreptococcales-Tissierellales* | *Peptostreptococcaceae* | *Filifactor* |
| d__Bacteria | *Firmicutes* | *Bacilli* | *Staphylococcales* | *Staphylococcaceae* | *Staphylococcus* |
| d__Bacteria | *Proteobacteria* | *Gammaproteobacteria* | *Burkholderiales* | *Comamonadaceae* | NA |
| d__Bacteria | *Actinobacteriota* | *Actinobacteria* | *Propionibacteriales* | *Propionibacteriaceae* | *Cutibacterium* |
| d__Bacteria | *Proteobacteria* | *Gammaproteobacteria* | *Enterobacterales* | *Enterobacteriaceae* | NA |
| d__Bacteria | *Fusobacteriota* | *Fusobacteriia* | *Fusobacteriales* | *Fusobacteriaceae* | *Fusobacterium* |
| d__Bacteria | *Proteobacteria* | *Gammaproteobacteria* | *Pseudomonadales* | *Pseudomonadaceae* | *Pseudomonas* |
| d__Bacteria | *Cyanobacteria* | *Cyanobacteriia* | *Chloroplast* | *Chloroplast* | *Chloroplast* |
| d__Bacteria | *Proteobacteria* | *Alphaproteobacteria* | *Sphingomonadales* | *Sphingomonadaceae* | *Novosphingobium* |
| d__Bacteria | *Proteobacteria* | *Gammaproteobacteria* | *Enterobacterales* | *Alteromonadaceae* | *Alishewanella* |
| d__Bacteria | *Actinobacteriota* | *Actinobacteria* | *Micrococcales* | *Intrasporangiaceae* | NA |
| d__Bacteria | *Proteobacteria* | *Alphaproteobacteria* | *Caulobacterales* | *Caulobacteraceae* | *Brevundimonas* |
| d__Bacteria | *Bacteroidota* | *Bacteroidia* | *Bacteroidales* | *Prevotellaceae* | *Prevotella* |
| d__Bacteria | *Actinobacteriota* | *Actinobacteria* | *Corynebacteriales* | *Corynebacteriaceae* | *Corynebacterium* |
| d__Bacteria | *Firmicutes* | *Clostridia* | *Lachnospirales* | *Lachnospiraceae* | *Lachnoanaerobaculum* |
| d__Bacteria | *Actinobacteriota* | *Actinobacteria* | *Corynebacteriales* | *Corynebacteriaceae* | *Corynebacterium* |
| d__Bacteria | *Proteobacteria* | *Gammaproteobacteria* | *Burkholderiales* | *Oxalobacteraceae* | *Herbaspirillum* |
| d__Bacteria | *Bacteroidota* | *Bacteroidia* | *Flavobacteriales* | *Weeksellaceae* | *Cloacibacterium* |
| d__Bacteria | *Proteobacteria* | *Gammaproteobacteria* | *Pseudomonadales* | *Moraxellaceae* | *Acinetobacter* |
| d__Bacteria | *Proteobacteria* | *Gammaproteobacteria* | *Xanthomonadales* | *Xanthomonadaceae* | *Stenotrophomonas* |
| d__Bacteria | *Proteobacteria* | *Gammaproteobacteria* | *Burkholderiales* | *Comamonadaceae* | NA |
| d__Bacteria | *Proteobacteria* | *Gammaproteobacteria* | *Burkholderiales* | *Comamonadaceae* | NA |
| d__Bacteria | *Proteobacteria* | *Gammaproteobacteria* | *Burkholderiales* | *Alcaligenaceae* | NA |
| d__Bacteria | *Actinobacteriota* | *Actinobacteria* | *Micrococcales* | *Micrococcaceae* | *Rothia* |
| d__Bacteria | *Gemmatimonadota* | *Gemmatimonadetes* | *Gemmatimonadales* | *Gemmatimonadaceae* | *uncultured* |
| d__Bacteria | *Proteobacteria* | *Gammaproteobacteria* | *Enterobacterales* | *Idiomarinaceae* | *Aliidiomarina* |
| d__Bacteria | *Proteobacteria* | *Alphaproteobacteria* | *Sphingomonadales* | *Sphingomonadaceae* | *Sphingobium* |
| d__Bacteria | *Proteobacteria* | *Gammaproteobacteria* | *Burkholderiales* | *Burkholderiaceae* | *Cupriavidus* |
| d__Bacteria | *Bacteroidota* | *Bacteroidia* | *Bacteroidales* | *Porphyromonadaceae* | *Porphyromonas* |
| d__Bacteria | *Actinobacteriota* | *Actinobacteria* | *0319-7L14* | *0319-7L14* | *0319-7L14* |
| d__Bacteria | *Proteobacteria* | *Gammaproteobacteria* | *Pseudomonadales* | *Pseudomonadaceae* | *Pseudomonas* |
| d__Bacteria | *Actinobacteriota* | *Actinobacteria* | *Micrococcales* | *Micrococcaceae* | NA |
| d__Bacteria | *Fusobacteriota* | *Fusobacteriia* | *Fusobacteriales* | *Fusobacteriaceae* | *Fusobacterium* |
| d__Bacteria | *Proteobacteria* | *Gammaproteobacteria* | *Pseudomonadales* | *Pseudomonadaceae* | *Pseudomonas* |

### Table S2. List of contaminants from the positive controls

The feature ID and feature taxonomy were assigned using the SILVA 132 database. 39 Features were identified as contaminants in *decontam* package. The contaminants were more prevalent in positive controls (environmental controls and curette wash) than in supragingival plaque samples.

| **Kingdom** | **Phylum** | **Class** | **Order** | **Family** | **Genus** |
| --- | --- | --- | --- | --- | --- |
| d__Bacteria | *Bacteroidota* | *Bacteroidia* | *Bacteroidales* | *Prevotellaceae* | *NA* |
| d__Bacteria | *Proteobacteria* | *Gammaproteobacteria* | *Pseudomonadales* | *Moraxellaceae* | *Acinetobacter* |
| d__Bacteria | *Proteobacteria* | *Gammaproteobacteria* | *Pseudomonadales* | *Pseudomonadaceae* | *NA* |
| d__Bacteria | *Proteobacteria* | *Gammaproteobacteria* | *Pseudomonadales* | *Pseudomonadaceae* | *Pseudomonas* |
| d__Bacteria | *Fusobacteriota* | *Fusobacteriia* | *Fusobacteriales* | *Fusobacteriaceae* | *Fusobacterium* |
| d__Bacteria | *Desulfobacterota* | *Desulfovibrionia* | *Desulfovibrionales* | *Desulfoplanaceae* | *Desulfoplanes* |
| d__Bacteria | *Proteobacteria* | *Gammaproteobacteria* | *Pseudomonadales* | *Pseudomonadaceae* | *Pseudomonas* |
| d__Bacteria | *Actinobacteriota* | *Actinobacteria* | *Corynebacteriales* | *Corynebacteriaceae* | *Corynebacterium* |
| d__Bacteria | *Proteobacteria* | *Gammaproteobacteria* | *Pseudomonadales* | *Moraxellaceae* | *Enhydrobacter* |
| d__Bacteria | *Proteobacteria* | *Gammaproteobacteria* | *Burkholderiales* | *Comamonadaceae* | *NA* |
| d__Bacteria | *Proteobacteria* | *Gammaproteobacteria* | *Pseudomonadales* | *Moraxellaceae* | *Acinetobacter* |
| d__Bacteria | *Proteobacteria* | *Gammaproteobacteria* | *Pseudomonadales* | *Moraxellaceae* | *Acinetobacter* |
| d__Bacteria | *Proteobacteria* | *Gammaproteobacteria* | *Burkholderiales* | *Burkholderiaceae* | *Burkholderia-Caballeronia-Paraburkholderia* |
| d__Bacteria | *Proteobacteria* | *Gammaproteobacteria* | *Burkholderiales* | *Comamonadaceae* | *NA* |
| d__Bacteria | *Proteobacteria* | *Gammaproteobacteria* | *Burkholderiales* | *Alcaligenaceae* | *Alcaligenes* |
| d__Bacteria | *Proteobacteria* | *Alphaproteobacteria* | *Caulobacterales* | *Caulobacteraceae* | *Brevundimonas* |
| d__Bacteria | *Proteobacteria* | *Alphaproteobacteria* | *Sphingomonadales* | *Sphingomonadaceae* | *NA* |
| d__Bacteria | *Actinobacteriota* | *Actinobacteria* | *Micrococcales* | *Micrococcaceae* | *Micrococcus* |
| d__Bacteria | *Proteobacteria* | *Gammaproteobacteria* | *Burkholderiales* | *Oxalobacteraceae* | *Herminiimonas* |
| d__Bacteria | *Proteobacteria* | *Alphaproteobacteria* | *Acetobacterales* | *Acetobacteraceae* | *Acidocella* |
| d__Bacteria | *Proteobacteria* | *Gammaproteobacteria* | *NA* | *NA* | *NA* |
| d__Bacteria | *Bacteroidota* | *Bacteroidia* | *Bacteroidales* | *Prevotellaceae* | *Prevotella* |
| d__Bacteria | *Actinobacteriota* | *Actinobacteria* | *Propionibacteriales* | *Propionibacteriaceae* | *Cutibacterium* |
| d__Bacteria | *Proteobacteria* | *Gammaproteobacteria* | *Enterobacterales* | *Enterobacteriaceae* | NA |
| d__Bacteria | *Proteobacteria* | *Alphaproteobacteria* | *Rhizobiales* | *Xanthobacteraceae* | NA |
| d__Bacteria | *Proteobacteria* | *Gammaproteobacteria* | *Pseudomonadales* | *Pseudomonadaceae* | *Pseudomonas* |
| d__Bacteria | *Proteobacteria* | *Alphaproteobacteria* | *Sphingomonadales* | *Sphingomonadaceae* | NA |
| d__Bacteria | *Proteobacteria* | *Gammaproteobacteria* | *Pseudomonadales* | *Pseudomonadaceae* | *Pseudomonas* |
| d__Bacteria | *Proteobacteria* | *Gammaproteobacteria* | *Pseudomonadales* | *Pseudomonadaceae* | *Pseudomonas* |
| d__Bacteria | *Proteobacteria* | *Alphaproteobacteria* | *Caulobacterales* | *Caulobacteraceae* | *Brevundimonas* |
| d__Bacteria | *Firmicutes* | *Negativicutes* | *Veillonellales-Selenomonadales* | *Selenomonadaceae* | *Selenomonas* |
| d__Bacteria | *Proteobacteria* | *Gammaproteobacteria* | *Burkholderiales* | *Oxalobacteraceae* | *Herbaspirillum* |
| d__Bacteria | *Bacteroidota* | *Bacteroidia* | *Flavobacteriales* | *Weeksellaceae* | *Cloacibacterium* |
| d__Bacteria | *Proteobacteria* | *Gammaproteobacteria* | *Burkholderiales* | *Comamonadaceae* | NA |
| d__Bacteria | *Bacteroidota* | *Bacteroidia* | *Flavobacteriales* | *Flavobacteriaceae* | *Flavobacterium* |
| d__Bacteria | *Firmicutes* | *Bacilli* | *Staphylococcales* | *Staphylococcaceae* | *Staphylococcus* |
| d__Bacteria | *Proteobacteria* | *Gammaproteobacteria* | *Pseudomonadales* | *Pseudomonadaceae* | *Pseudomonas* |
| d__Bacteria | *Proteobacteria* | *Gammaproteobacteria* | *Methylococcales* | *Methylomonadaceae* | NA |

### Table S3. Participant characteristics of the analysed cohort (n=100) and overall participants in the study (n=280)

| **Variable** | **Categories** | **Analysed n (%)** 100 | **Overall n (%)** 280 |
| --- | --- | --- | --- |
| **Sociodemographic variable** | | | |
| Age (Mean SD) |  | 41.92 (13.37) | 44.85 (15.08) |
| Sex (F:M) | Female:Male | 63:37 | 164:116 |
| Self-identification | Aboriginal | 91 (91.0) | 244 (89.1) |
|  | Torres Strait Islander | 0 | 3 (1.1) |
|  | Both | 6 (6.0) | 11 (4.0) |
|  | Other Indigenous groups | 3 (3.0) | 16 (5.8) |
| Age category | > 55 years | 18 (19.0) | 78 (28.2) |
|  | 18-34 years | 34 (34.0) | 73 (26.4) |
|  | 35-54 years | 48 (47.0) | 126 (45.5) |
| Data collection location | Clinical/Dental Facility | 44 (44.4) | 126 (45.3) |
|  | Home/Community Centre | 55 (55.6) | 152 (54.7) |
| **Individual level socioeconomic variables** | | | |
| Education | Secondary education or less | 58 (58.0) | 157 (57.1) |
|  | Tertiary education | 42 (42.0) | 118 (42.9) |
| Income Source | Job | 61 (61) |  |
|  | Centrelink payment | 39 (39) |  |
| Healthcare card | No | 51 (51.0) | 124 (44.3) |
|  | Yes | 49 (49.0) | 156 (55.7) |
| Difficulty in paying $100 for dental treatment | Hard | 76 (76.0) | 213 (77.5) |
|  | Not hard | 24 (24.0) | 62 (22.5) |
| **Neighbourhood level socioeconomic variables** | | | |
| Remoteness | Metropolitan | 86 (86.0) | 218 (80.1) |
|  | Regional | 14 (14.0) | 54 (19.9) |
| SEIFA Index | High SES | 41 (41.0) | 107 (38.8) |
|  | Low SES | 59 (59.0) | 169 (61.2) |
| **Knowledge, attitudes, and beliefs variables** | | | |
| Last dental visit | Less than one year ago | 34 (34.0) | 84 (30.7) |
|  | More than one year ago | 66 (66.0) | 190 (69.3) |
| Reason for visiting dentist | Check-up | 20 (20.2) | 57 (20.9) |
|  | Problem | 79 (79.8) | 216 (79.1) |
| **Health Behaviours** | | | |
| Self-reported Diabetes | Present | 21/100 | 54 (100.0) |
| Smoking | Current Smoker | 36 (36.0) | 105 (38.6) |
|  | Non-Smoker | 38 (38.0) | 106 (39.0) |
|  | Past Smoker | 26 (26.0) | 61 (22.4) |
| Alcohol consumption | Yes: No | 61:39 | 161:112 |
| Frequency of Alcohol consumption | Monthly | 37 (37.0) | 91 (33.3) |
|  | Never | 39 (39.0) | 112 (41.0) |
|  | Weekly | 24 (24.0) | 70 (25.6) |
| **Oral health outcome variables** | | | |
| Self-rated oral health | Good | 29 (29.0) | 81 (29.5) |
|  | Poor | 71 (71.0) | 194 (70.5) |
| Dental caries | Absent | 42 (42.0) | 139 (49.6) |
|  | Present | 58 (58.0) | 141 (50.4) |

### Table S4. Alpha diversity test results for observed feature metrics and Shannon’s diversity metrics from plaque and saliva samples

| **Variable** | **Group 1** | **Group 2** | **Plaque samples** | | | | **Saliva Samples** | | | |
| --- | --- | --- | --- | --- | --- | --- | --- | --- | --- | --- |
|  |  |  | **Observed features** | | **Shannon index** | | **Observed features** | | **Shannon index** | |
|  |  |  | **W^†^ / Z ^¶^ Value** | ***p* value** | **W^†^ / Z ^¶^ Value** | ***p* value** | **W^†^ / Z ^¶^ Value** | ***p* value** | **W^†^ / Z ^¶^ Value** | ***p* value** |
| **Sociodemographic variables** | |  |  |  |  |  |  |  |  |  |
| Age | 18-34 years | 35-54 years | 0.64 | 0.25 | 0.23 | 0.40 | 0.82 | 0.20 | 0.42 | 0.33 |
|  | **18-34 years** | >55 years | 2.41 | **<0.01**** | 2.00 | **0.02*** | 2.44 | **<0.01**** | 0.66 | 0.25 |
|  | **35-54 years** | >55 years | 2.00 | **0.02*** | 1.92 | **0.02*** | 1.92 | **0.02*** | 0.35 | 0.36 |
|  | All group ^§^ |  | 6.08 | **0.04*** | 4.63 | 0.09 | 6.13 | **0.04*** | 0.45 | 0.79 |
| Gender | Male (32) | Female (60) | 1196 | 0.83 | 1401 | 0.09 | 1238 | 0.51 | 1315 | 0.22 |
| **Individual Level Socioeconomic variables** | | |  |  |  |  |  |  |  |  |
| Level of education | Tertiary (56) | **Secondary education or less** | 1627.5 | **<0.01**** | 1584 | **0.01*** | 1730.5 | **<0.01**** | 1593 | **<0.01**** |
| Income source | **Centrelink Payment** | Job | 1207.5 | 0.47 | 1132 | 0.87 | 1478.5 | **0.02*** | 1544 | **<0.01**** |
| Health care card | **Yes** | No | 1289 | 0.78 | 1418 | 0.24 | 889 | **0.01*** | 888 | **0.01*** |
| Difficulty in paying $100 for dental treatment | Hard | Not Hard | 1289 | 0.07 | 747 | 0.18 | 725 | 0.15 | 878 | 0.86 |
| **Neighbourhood Level Socioeconomic variables** | | |  |  |  |  |  |  |  |  |
| Remoteness | Metropolitan | Regional | 490 | 0.27 | 612 | 0.92 | 642 | 0.91 | 660 | 0.77 |
| SEIFA | All group |  | 2.60 | 0.45 | 2.97 | 0.39 | 1.34 | 0.71 | 4.03 | 0.25 |
| **Knowledge, attitudes, and beliefs variables** | | | | |  |  |  |  |  |  |
| Last Dental Visit | Less than a year ago | More than a year ago | 933 | 0.17 | 1046 | 0.58 | 978.5 | 0.35 | 1106 | 0.99 |
| Reason for dental visit | Check up | Problem | 769 | 0.85 | 697 | 0.42 | 793 | 0.91 | 863 | 0.46 |
| **Health behaviour variables** | |  |  |  |  |  |  |  |  |  |
| Smoking | **Current Smokers** | Non-Smoker | 1.17 | 0.11 | 0.17 | 0.42 | 2.06 | **0.01*** | 1.48 | 0.06 |
|  | **Current Smokers** | Past Smokers | 1.28 | 0.09 | 0.77 | 0.21 | 2.04 | **0.02*** | 1.93 | **0.02*** |
|  | Past Smokers | Non-Smokers | 0.22 | 0.41 | 0.62 | 0.26 | 0.19 | 0.42 | 0.61 | 0.27 |
|  | Overall^§^ |  | 2.08 | 0.35 | 0.58 | 0.74 | 5.80 | **0.05*** | 4.20 | 0.12 |
| Alcohol Consumption | Yes | No | 1266.5 | 0.58 | 1352 | 0.25 | 1293 | 0.33 | 1373 | 0.12 |
| **Oral health outcome variable** | |  |  |  |  |  |  |  |  |  |
| Self-rated oral health | Good | Poor | 877 | 0.24 | 963 | 0.61 | 1030 | 0.78 | 973 | 0.87 |
| Dental Caries | **Present** | Absent | 963.5 | 0.07 | 971 | 0.08 | 882.5 | **0.02** | 766 | **<0.01**** |

Bold variables indicate alpha diversity metrics that are both numerically higher and statistically significantly different between comparison groups, as indicated by p-values.

† Pairwise non-parametric Wilcoxon rank sum test was used to calculate the W statistics and *p-*value. § For multiple group comparison, the Kruskal Wallis rank sum test was used to calculate the chi-squared statistics and p values. ¶ This was followed by post-hoc Dunn’s test for pairwise comparison to calculate the Z statistics and p values. The level of significance * equal to 5% (*p*<0.05) and ** being equal to 1% (*p*<0.01).

### Table S5. Beta diversity using significance test results using Aitchison distances for plaque and saliva samples

| **Variable** | **Plaque samples** | | | **Saliva samples** | | |
| --- | --- | --- | --- | --- | --- | --- |
|  | **F-value** | ***R2*** | ***p-value*** | **F-value** | ***R2*** | ***p-value*** |
| **Age** | 2.69 | 0.052 | **<0.01**** | 1.94 | 0.038 | **<0.01**** |
| Gender | 0.81 | 0.008 | 0.64 | 1.00 | 0.010 | 0.38 |
| **Education** | 1.74 | 0.017 | **0.04*** | 2.02 | 0.020 | **<0.01**** |
| **Income source** | 1.90 | 0.037 | **0.01*** | 1.90 | 0.038 | **<0.01**** |
| **Healthcare card ownership** | 1.65 | 0.032 | **0.02*** | 1.42 | 0.020 | 0.07 |
| Paying $100 for dental treatment | 1.32 | 0.013 | 0.15 | 1.15 | 0.011 | 0.24 |
| **Remoteness** | 1.84 | 0.018 | **0.02*** | 1.16 | 0.011 | 0.23 |
| SEIFA | 1.27 | 0.028 | 0.61 | 0.96 | 0.009 | 0.46 |
| Last dental visit | 0.91 | 0.009 | 0.54 | 1.79 | 0.018 | **0.02*** |
| Reason for dental visit | 0.79 | 0.008 | 0.71 | 0.85 | 0.008 | 0.64 |
| **Smoking history** | 2.32 | 0.045 | **<0.01**** | 2.85 | 0.056 | **<0.01**** |
| Alcohol consumption | 1.03 | 0.010 | 0.35 | 1.00 | 0.010 | 0.39 |
| **Self-rated oral health** | 3.03 | 0.03 | **0.003**** | 2.32 | 0.023 | **<0.01**** |
| Dental caries | 1.47 | 0.014 | 0.09 | 2.28 | 0.022 | **<0.01**** |

The R^2^ represents the proportion of variance explained by the variable. The level of significance is * equal to 5% (*p*<0.05), and ** is equal to 1% (*p*<0.01).

###

### Table S6. Results from differential abundance analysis for the secondary level of education (ref. Tertiary education)

| **Feature** | **Coefficient** | **Standard error** | ***p-value*** | **Q-value** |
| --- | --- | --- | --- | --- |
| *F0058* | 1.36 | 0.29 | 0.00 | 0.00 |
| *Veillonella* | 1.25 | 0.16 | 0.00 | 0.03 |
| *Rikenellaceae RC9 gut group* | 1.05 | 0.38 | 0.00 | 0.04 |
| *Treponema* | 1.01 | 0.23 | 0.00 | 0.06 |
| *Palleniella* | 0.86 | 0.20 | 0.00 | 0.08 |
| *Lentimicrobium* | 0.82 | 0.36 | 0.01 | 0.09 |
| *Johnsonella* | 0.76 | 0.32 | 0.01 | 0.15 |
| *Lactobacillales order* | 0.67 | 0.19 | 0.01 | 0.16 |
| *Filifactor* | 0.58 | 0.34 | 0.02 | 0.20 |
| *Pasteurellaceae family* | 0.49 | 0.28 | 0.03 | 0.20 |
| *Centipeda* | -0.36 | 0.23 | 0.03 | 0.21 |
| *Oribacterium* | -0.45 | 0.21 | 0.03 | 0.22 |
| *Clostridia_vadinBB60_group* | -0.47 | 0.41 | 0.04 | 0.22 |
| *Streptococcus* | -0.56 | 0.17 | 0.04 | 0.24 |
| *Spirochaetaceae family* | -0.57 | 0.52 | 0.04 | 0.24 |
| *Eubacterium brachy group* | -0.63 | 0.29 | 0.05 | 0.24 |

### Table S7. Results from differential abundance analysis for income source as Centrelink (ref. Job)

| **Feature** | **Coefficient** | **Standard error** | ***p-value*** | **Q-value** |
| --- | --- | --- | --- | --- |
| *Scardovia* | 1.29 | 0.50 | 0.01 | 0.13 |
| *F0058* | 0.95 | 0.30 | 0.00 | 0.05 |
| *Filifactor* | 0.88 | 0.34 | 0.01 | 0.13 |
| *Eubacterium* | 0.87 | 0.29 | 0.00 | 0.06 |
| *Rikenellaceae_RC9_gut_group* | 0.80 | 0.39 | 0.04 | 0.23 |
| *Lachnospiraceae family* | 0.72 | 0.37 | 0.05 | 0.24 |
| *Rothia* | 0.68 | 0.19 | 0.00 | 0.01 |
| *Treponema* | 0.55 | 0.23 | 0.02 | 0.15 |
| *Catonella* | 0.50 | 0.22 | 0.02 | 0.18 |
| *Corynebacterium* | 0.43 | 0.20 | 0.03 | 0.19 |
| *Palleniella* | -0.44 | 0.20 | 0.03 | 0.19 |
| *Solobacterium* | -0.45 | 0.23 | 0.05 | 0.25 |
| *Alloprevotella* | -0.61 | 0.21 | 0.00 | 0.09 |
| *Clostridia_UCG* | -0.61 | 0.30 | 0.04 | 0.23 |
| *Bergeyella* | -0.78 | 0.24 | 0.00 | 0.04 |
| *Neisseria* | -0.79 | 0.36 | 0.03 | 0.19 |
| *Aggregatibacter* | -0.85 | 0.38 | 0.03 | 0.19 |
| *Pasteurellaceae family* | -1.31 | 0.27 | 0.00 | 0.00 |

### Table S8. Results from differential abundance analysis for the presence of dental caries (ref. no caries)

| **Feature** | **Coefficient** | **Standard error** | ***p-value*** | **Q-value** |
| --- | --- | --- | --- | --- |
| *Rikenellaceae RC9 gut group* | 1.44 | 0.37 | 0.00 | 0.01 |
| *Actinomyces* | -0.55 | 0.16 | 0.00 | 0.02 |
| *Peptoanaerobacter* | 1.38 | 0.41 | 0.00 | 0.03 |
| *Anaeroglobus* | 1.06 | 0.33 | 0.00 | 0.03 |
| *Phocaeicola* | 1.24 | 0.40 | 0.00 | 0.04 |
| *Filifactor* | 1.02 | 0.33 | 0.00 | 0.04 |
| *Prevotellaceae UCG001* | 1.00 | 0.34 | 0.00 | 0.06 |
| *Veillonella* | -0.48 | 0.17 | 0.00 | 0.06 |
| *F0058* | 0.83 | 0.30 | 0.01 | 0.09 |
| *Atopobium* | -0.56 | 0.21 | 0.01 | 0.10 |
| *Clostridia UCG* | -0.78 | 0.29 | 0.01 | 0.10 |
| *Treponema* | 0.55 | 0.23 | 0.01 | 0.15 |
| *Fretibacterium* | 0.63 | 0.28 | 0.02 | 0.19 |
| *Lactobacillales order* | -0.44 | 0.19 | 0.02 | 0.19 |
| *Clostridia_vadinBB60_group* | 0.90 | 0.41 | 0.03 | 0.20 |
| *Porphyromonas* | 0.46 | 0.21 | 0.03 | 0.20 |
| *Lachnospiraceae family* | 0.75 | 0.36 | 0.04 | 0.20 |
| *Solobacterium* | -0.46 | 0.22 | 0.04 | 0.20 |
| *Megasphaera* | -0.48 | 0.23 | 0.04 | 0.20 |
| *Catonella* | 0.42 | 0.22 | 0.05 | 0.22 |
| *Eubacterium brachy group* | 0.55 | 0.29 | 0.06 | 0.24 |

### Table S9. Results from the mediation analysis of income source and education as the exposure and dental caries as the outcome.

| **Variables** | **Direct Effect**  **Mean (SE)** | **Mediation Effect**  **Mean (SE)** | **Total Effect**  **Mean (SE)** |
| --- | --- | --- | --- |
| Income source (Centrelink payment) | 0.09 (0.26) | 0.28 (0.32) | 0.37 (0.05) |
| Education level (Secondary education or less) | 0.10 (0.01) | 0.03 (0.02) | 0.14 (0.03) |

### Table S10. Component-wise mediation effect of individual microbial taxa on the relationship between secondary education and dental caries.

| **Feature** | **Mean** | **LCI** | **UCI** |
| --- | --- | --- | --- |
| *Streptococcus* | 0.07 | -0.06 | 0.19 |
| *Neisseria* | 0.06 | -0.02 | 0.13 |
| *Veillonella* | 0.02 | -0.03 | 0.07 |
| *Megasphaera* | 0.00 | -0.01 | 0.00 |
| *Palleniella* | -0.01 | -0.02 | 0.01 |
| *Prevotella* | -0.03 | -0.06 | 0.00 |
| *Fusobacterium* | -0.03 | -0.11 | 0.05 |
| *Pasteurellaceae family* | -0.03 | -0.07 | 0.01 |

LCI: Lower confidence interval; UCI: Upper confidence interval

### Table S11. Component-wise mediation effect of individual microbial taxa on the relationship between Centrelink income and dental caries.

| **Feature** | **Mean** | **LCI** | **UCI** |
| --- | --- | --- | --- |
| *Veillonella* | 0.14 | -0.26 | 0.54 |
| *Pasteurellaceae family* | 0.11 | -0.50 | 0.73 |
| *Streptococcus* | 0.10 | -0.11 | 0.31 |
| *Prevotella* | 0.06 | -0.26 | 0.38 |
| *Palleniella* | 0.04 | -0.20 | 0.28 |
| *Megasphaera* | -0.03 | -0.18 | 0.12 |
| *Streptococcus* | -0.03 | -0.10 | 0.04 |
| *Fusobacterium* | -0.07 | -0.23 | 0.09 |

LCI: Lower confidence interval; UCI: Upper confidence interval.
